# Multiple *cis*-regulatory elements control *prox1a* expression in distinct lymphatic vascular beds

**DOI:** 10.1242/dev.202525

**Published:** 2024-05-09

**Authors:** Virginia Panara, Hujun Yu, Di Peng, Karin Staxäng, Monika Hodik, Beata Filipek-Gorniok, Jan Kazenwadel, Renae Skoczylas, Elizabeth Mason, Amin Allalou, Natasha L. Harvey, Tatjana Haitina, Benjamin M. Hogan, Katarzyna Koltowska

**Affiliations:** ^1^ Department of Immunology, Genetics and Pathology, Uppsala University, Uppsala 75185, Sweden; ^2^ Beijer Gene and Neuro Laboratory, Uppsala University, Uppsala 75185, Sweden; ^3^ Organogenesis and Cancer Program, Peter MacCallum Cancer Centre, Melbourne, VIC 3000, Australia; ^4^ Sir Peter MacCallum Department of Oncology and Department of Anatomy and Physiology, University of Melbourne, Melbourne, VIC 3000, Australia; ^5^ BioVis Core Facility, Platform EM, Uppsala University, Uppsala 75185, Sweden; ^6^ Centre for Cancer Biology, University of South Australia and SA Pathology, Adelaide, South Australia 5001, Australia; ^7^ Uppsala University, Department of Information Technology, Division of Visual Information and Interaction, and SciLifeLab BioImage Informatics Facility, Uppsala University, Uppsala 75185, Sweden; ^8^ Adelaide Medical School, University of Adelaide, Adelaide, South Australia 5005, Australia; ^9^ Department of Organismal Biology, Uppsala University, Uppsala 75236, Sweden

**Keywords:** Prox1, Enhancers, Evolutionary conservation, Gene regulation, Lymphatic endothelial cell, Transcription factor, Zebrafish

## Abstract

During embryonic development, lymphatic endothelial cell (LEC) precursors are distinguished from blood endothelial cells by the expression of Prospero-related homeobox 1 (Prox1), which is essential for lymphatic vasculature formation in mouse and zebrafish. *Prox1* expression initiation precedes LEC sprouting and migration, serving as the marker of specified LECs. Despite its crucial role in lymphatic development, *Prox1* upstream regulation in LECs remains to be uncovered. SOX18 and COUP-TFII are thought to regulate *Prox1* in mice by binding its promoter region. However, the specific regulation of *Prox1* expression in LECs remains to be studied in detail. Here, we used evolutionary conservation and chromatin accessibility to identify enhancers located in the proximity of zebrafish *prox1a* active in developing LECs. We confirmed the functional role of the identified sequences through CRISPR/Cas9 mutagenesis of a lymphatic valve enhancer. The deletion of this region results in impaired valve morphology and function. Overall, our results reveal an intricate control of *prox1a* expression through a collection of enhancers. Ray-finned fish-specific distal enhancers drive pan-lymphatic expression, whereas vertebrate-conserved proximal enhancers refine expression in functionally distinct subsets of lymphatic endothelium.

## Teaser

**Summary:** Although *Prox1* is a key factor in lymphatic endothelial cell development, little is known about its *cis*-regulation. Here, we characterise multiple *prox1a* enhancers active in subsets of zebrafish lymphatic vasculature.

## INTRODUCTION

Transcription factor (TF) regulation plays a pivotal role in cell specification and the acquisition of tissue identity during development (Spitz and Furlong, 2012). The TF PROX1 is involved in lymphatic vasculature development. In mouse, PROX1 is expressed by the lymphatic progenitors and is the first marker of specified lymphatic endothelial cells (LECs) (Wigle and Oliver, 1999). *Prox1* mutants are characterised by the loss of all lymphatic structures (Wigle and Oliver, 1999). However, during embryonic development, *prox1* is expressed in multiple tissues, such as the central nervous system, liver, retina and skeletal muscles (Glasgow and Tomarev, 1998; Pistocchi et al., 2008), implying the necessity for mechanisms restricting its expression in a tissue-specific manner. Although TFs regulating *Prox1* expression in the lymphatics have been described in mammals (François et al., 2008; Srinivasan et al., 2010), the upstream *cis-*regulation of *Prox1* remains largely unexplored, leaving a comprehensive understanding of the regulatory logic of the gene incomplete.

Enhancers are *cis-*regulatory elements that can be located proximally or distally to a gene locus. Enhancers can function in a modular fashion, with tissue-specific enhancers activated independently (Long et al., 2016). Enhancer regulation is particularly interesting in a developmental context, as many ‘developmental toolbox’ genes are differentially regulated through enhancer activity. Important enhancer elements active in lymphatic and blood vascular development have been identified in genes such as *gata2a* (Shin et al., 2019), *flt1* (Bussmann et al., 2010), *notch1b* (Chiang et al., 2017), *fli1* (Villefranc et al., 2007) and *etsrp* (Veldman and Lin, 2012)*.* However, to date only one lymphatic-specific *Prox1* enhancer (Kazenwadel et al., 2023), with activity enriched in the lymphatic valves, has been described. Therefore, the number, contextual nature and identity of the enhancers driving *Prox1* expression in lymphatics remains to be investigated.

In zebrafish, the most studied lymphatic beds are the trunk and facial lymphatics (Eng et al., 2019; Hogan et al., 2009; Küchler et al., 2006; Okuda et al., 2012; Yaniv et al., 2006) which require *prox1* for correct development (Grimm et al., 2023). As a result of the teleost-specific genome duplication event, two zebrafish co-orthologues of mammalian *Prox1* have been described in the literature, called respectively *prox1a* and *prox3* (previously referred to as *prox1b*) (Giacco et al., 2010). *prox1a* is an early marker of lymphatic identity, being expressed by lymphatic progenitors in the posterior cardinal vein (PCV) at 32 h post-fertilization (hpf), before the onset of sprouting (Dunworth et al., 2014; Koltowska et al., 2015a). Its expression marks all described lymphatic beds for the duration of larval development. Mutants for *prox1a* and *prox3* show a reduction in the number of LECs (Grimm et al., 2023), confirming the importance of these factors for LEC development in zebrafish. In particular, it is *prox1a* that has a prominent developmental role in zebrafish lymphangiogenesis (Grimm et al., 2023; Koltowska et al., 2015a). Despite this, little is known about *prox1a* regulation. Although the TFs Coup-TFII (Nr2f2) and Sox18 do not seem to regulate *prox1a* (van Impel et al., 2014), a role of Vegfc signalling upstream of *prox1a* in the specification of LECs has been reported (Koltowska et al., 2015a). However, the *cis*-regulatory elements of *prox1a* and their evolution are still to be described.

In this study, we aimed to characterise the enhancers regulating the expression of *prox1a* in the developing lymphatics. We identified several elements driving expression in different subsets of the lymphatic endothelium. These include both conserved elements across vertebrates and elements specific to actinopterygians. Our results suggest that *prox1a* is tightly regulated in a spatially patterned manner by a cohort of enhancers acting in concert.

## RESULTS

### The *prox1a* locus is enriched in evolutionarily conserved non-coding regions

**Fig. 1.**
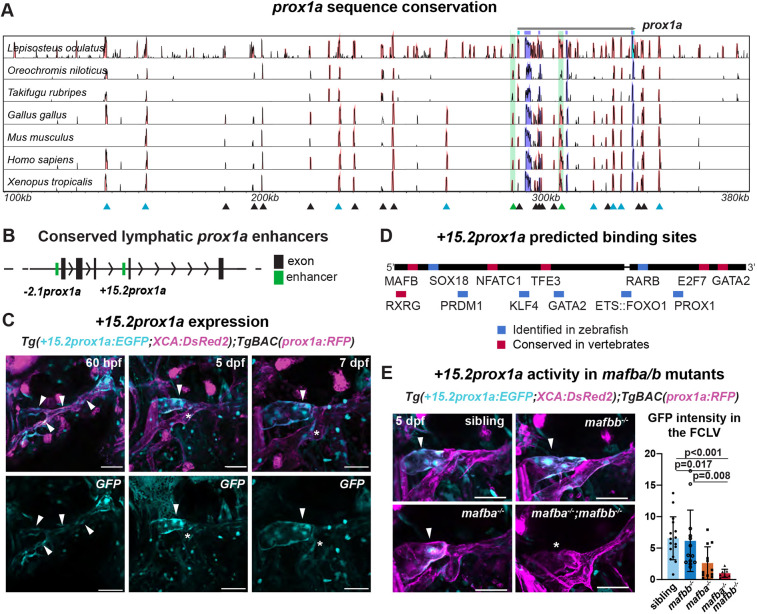
**The conserved +*15.2prox1a* enhancer drives expression in a subset of the facial lymphatics.** (A) Conservation analysis of the 380 kbp region surrounding the zebrafish *prox1a* locus compared with seven vertebrate species. Blue peaks, exons; red peaks, conserved non-coding DNA; black arrowheads, conserved peaks; blue arrowheads, tested peaks; green arrowheads, identified −*2.1prox1a* and +*15.2prox1a* lymphatic enhancers. In the 5′ the first 100 kbp of the alignment contain no conservation peak and has been omitted from the graph. (B) *prox1a* locus showing the position of the identified conserved lymphatic enhancers. Green boxes, −*2.1prox1a* and +*15.2prox1a* enhancers; black boxes, exons. (C) Confocal projections of facial lymphatics labelled with *Tg(*+*15.2prox1a:EGFP; XCA:DsRed2)^uu7kk^* (cyan) and *Tg(prox1a:RFP)^nim5^* (magenta) at 60 hpf, 5 dpf and 7 dpf. Arrowheads show expression in the facial LECs (60 hpf) and FCLV (5 and 7 dpf). Asterisks show expression in facial lymphatic endothelium. (D) Predicted endothelial TF binding sites in +*15.2prox1a*. Blue, binding sites identified in zebrafish (*P*<1e-04); red, conserved binding sites within vertebrates. (E) Quantification of +*15.2prox1a* activity in *mafba*/*b* mutants. Left: confocal projections of facial lymphatics labelled with *Tg(*+*15.2prox1a:EGFP; XCA:DsRed2)^uu7kk^* (cyan) and *Tg(prox1a:RFP)^nim5^* (magenta) at 5 dpf showing enhancer activity (arrowhead) or lack thereof (asterisk) in the FCLV. Right: quantification of signal intensity in the FCLV normalised to the ganglia in 5 dpf embryos. Sibling (*n*=16) versus *mafbb*^−*/*−^ (*n*=13), *mafba*^−*/*−^ (*n*=12) and *mafba*^−*/*−^*;mafbb*^−*/*−^ (*n*=7). Mean±s.d. Sibling versus *mafbb*^−*/*−^, not significant (ns) (*P*>0.999); sibling versus *mafba*^−*/*−^, *P*=0.017; sibling versus *mafba*^−*/*−^*;mafbb*^−*/*−^, *P*<0.001; *mafbb*^−*/*−^ versus *mafba*^−*/*−^, ns (*P*=0.146); *mafbb*^−*/*−^ versus *mafba*^−*/*−^*;mafbb*^−*/*−^, *P*=0.008; *mafba*^−*/*−^ versus *mafba*^−*/*−^*;mafbb*^−*/*−^, ns (*P*>0.999) (Kruskal–Wallis test with Dunn's multiple comparison test). Four technical replicates, biological replicates correspond to the number of data points per condition. Scale bars: 50 μm.

To identify enhancer elements in the zebrafish *prox1a* locus, we analysed DNA conservation, as conserved non-coding elements (CNE) close to a gene can indicate the presence of enhancers. We aligned the region of the *PROX1/prox1a* locus of seven Osteichthyes species against that of the zebrafish using mVISTA (Fig. 1A; Table S1). The species were selected to have a balanced spread along the phylogeny. Local microsynteny, which is the conservation of the identity and position of the features surrounding the gene of interest, was verified by comparing the identities of neighbouring loci (Fig. S1A). Microsynteny indicates that no major genomic rearrangements, such as insertions, deletions or inversions of whole regions, have occurred around the considered locus, and therefore supports the presence of evolutionarily conserved enhancers. In all species analysed, an *SMYD2* homolog was located downstream of *prox1a*. Similarly, *RPS6KC1* was located upstream of the locus in all cases except zebrafish. Consequently, we defined the regions of interest for the conservation analysis as the sequences between the two loci adjacent to *PROX1/prox1a*, encompassing the long intergenic region upstream of *prox1a* (300 kbp). We recovered 25 conserved non-coding sequences, located upstream, downstream and in the intronic regions of *PROX1/prox1a* (Fig. 1A). We also investigated conservation in the other described *PROX1* co-orthologue, *prox3*. Interestingly, *prox3* presents very low levels of conservation outside of exon sequences, with only two Teleost-conserved elements identified in the analysis and none conserved with other vertebrates in intergenic regions (Fig. S1B).

**Fig. 2.**
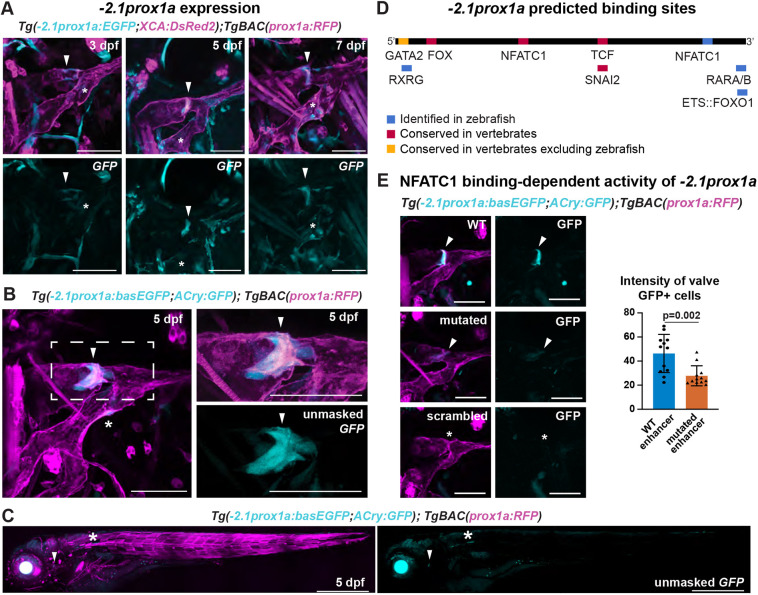
**The conserved −*2.1prox1a* enhancer drives expression in the lymphatic valve.** (A) Confocal projections of facial lymphatics labelled with *Tg(*−*2.1prox1a:EGFP; XCA:DsRed2)^uu3kk^* (cyan) and *Tg(prox1a:RFP)^nim5^* (magenta) at 3, 5 and 7 dpf. Arrowheads show expression in the developing lymphatic valve. Asterisks show expression in the facial lymphatic endothelium. (B) Confocal projections of the facial lymphatics labelled with *Tg(*−*2.1prox1a:basEGFP;ACry:GFP)^uu10kk^* (cyan) and *Tg(prox1a:RFP)^nim5^* (magenta) at 5 dpf. Left: expression in the lymphatic valve (arrowhead) and facial lymphatic endothelium (asterisk). Right: magnification of the boxed area in the left panel. (C) Confocal projection of the whole embryo at 5 dpf labelled with *Tg(*−*2.1prox1a:basEGFP;ACry:GFP)^uu10kk^* (cyan) and *Tg(prox1a:RFP)^nim5^* (magenta). Arrowhead shows expression in the lymphatic valve. Asterisks show additional expression in the skin. (D) Predicted endothelial TF binding sites in −*2.1prox1a*. Blue, binding sites identified in zebrafish (*P*<1e-04); red, conserved binding sites within vertebrates; yellow, binding sites conserved in vertebrates but absent in zebrafish. (E)  Representative images and quantification of Nfatc1 binding-dependent −*2.1prox1a* activity. Left: confocal projections of lymphatic valve labelled with WT, Nfatc1 binding site-mutated or scrambled −*2.1prox1a:basEGFP;ACry:GFP* constructs. Arrowheads show signal in the valve. Asterisk shows missing signal in the valve. Right: quantification of signal intensity in the valve cells expressing GFP in 5 dpf injected embryos. Mean±s.d. WT (*n*=12) versus Nfatc1 binding site-mutated (*n*=12) injected embryos at 5 dpf; *P*=0.002 (two-tailed Mann–Whitney test). Four technical replicates, biological replicates correspond to the number of data points per condition. Scale bars: 50μm (A,B,E); 500 μm (C).

We then tested selected *prox1a* CNEs for regulatory activity. Histone modifications have been linked to non-coding elements such as promoters and enhancers. Specifically, H3K4me1 marks primed and active enhancers (Heintzman et al., 2007) and H3K27ac indicates active enhancers (Bonn et al., 2012; Creyghton et al., 2010). Currently, no LEC-specific zebrafish databases for H3K4me1 and H3K27ac are available. Whole-body databases were instead used to identified ten of the selected *prox1a* CNEs as primed enhancers (Aday et al., 2011; Bogdanović et al., 2012) (Fig. 1A; Fig. S1C). These were subsequently cloned into the Zebrafish Enhancer Detection (ZED) vector (Bessa et al., 2009) and tested *in vivo* in F1 (Table S2). Two of the tested sequences drove GFP expression in the subsets of lymphatic endothelium. The conservation of the identified elements was further confirmed using PhyloP and the Multiz alignment to vertebrate tracks in the UCSC Genome browser (Fig. S1D). Of the two elements, +*15.2prox1a* [located 15.2 kbp downstream of the transcription start site (TSS)] was enriched in the facial collecting lymphatic vessel (FCLV) (Fig. 1B,C; Fig. S1E; Table 1; Table S2), and −*2.1prox1a* (located 2.1 kbp upstream of the TSS) was enriched in the lymphatic valve (Figs 1B, 2A-C; Fig. S2A; Table 1; Table S2).

**Table 1.**
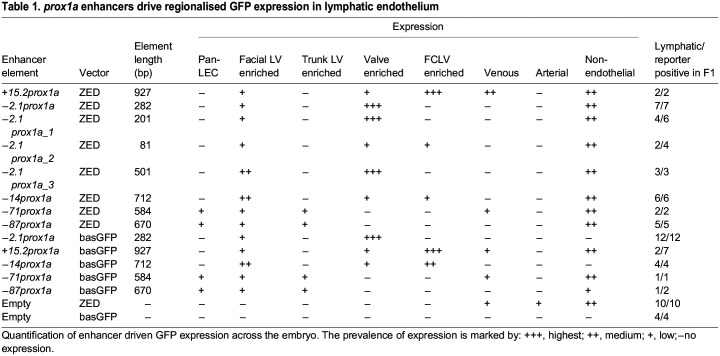
*prox1a* enhancers drive regionalised GFP expression in lymphatic endothelium

### +*15.2prox1a* drives expression in the FCLV

+*15.2prox1a* is a 927 bp element located in intron 3 of *prox1a* (Fig. 1B; Fig. S1C). The element is composed of two separate conservation peaks divided by 17 bp (Fig. 1D). At 5 and 7 days post-fertilization (dpf) the element is able to drive GFP expression in the FCLV, with faint additional expression in the facial lymphatics (Fig. 1C; Fig. S1E; Table 1). We also observed the reporter expression at 60 hpf in the developing facial lymphatic, but not in the ventral aorta lymphangioblast (VA-L) (Fig. 1C; Fig. S1E,F). In addition, +*15.2prox1a* is active in the venous endothelium in the primary head sinus (PHS) (Fig. S1G) and in the PCV (Fig. S1H). In a whole-body image, additional expression can be detected in the gallbladder and pancreas (Fig. S1I). As this element is highly conserved across vertebrates, we used a *P*-value cut-off of 1e-02 for our MEME Suite (Bailey and Elkan, 1994) and TOMTOM (Gupta et al., 2007) to determine the motifs and putative transcription factor binding sites present. We identified conserved binding sites for multiple known lymphatic regulators, such as Gata2, Nfatc1, Mafb, Sox18, Prox1 (Arnold et al., 2022; Geng et al., 2016; Kazenwadel et al., 2015; Koltowska et al., 2015b; Shin et al., 2019) and the retinoic acid receptors Rarb and Rxrg (Bowles et al., 2014; Marino et al., 2011). We also found putative sites for TFs which have been implied in regulation of *Prox1* expression or are related to endothelial cell (EC) identity such as Tfe3, Ets::Foxo1, Prdm1 and Klf4 (Dieterich et al., 2015; Niimi et al., 2019; Park et al., 2014; Pham et al., 2007; Tai-Nagara et al., 2020; De Val et al., 2008; Yoshimatsu et al., 2011) (Fig. 1D; Fig. S1J,K; Tables S4,S5). In order to test the functional importance of the predicted binding sites, we took advantage of the *mafba^uq4bh^* and *mafbb^ub47bh^* mutant lines available in our lab (Arnold et al., 2022; Koltowska et al., 2015b). Quantification of the activity of +*15.2prox1a* in the mutant lines revealed a 60% reduction in enhancer activity in *mafba* mutants and 85% in double mutants (Fig. 1E). This confirms the functional relevance of the MAFB predicted binding site for +*15.2prox1a* activity, and suggests this enhancer is an important driver of *prox1a* contributing to the regulatory logic necessary for its correct spatial expression in developing lymphatics.

### −*2.1prox1a* drives expression in the lymphatic valve across developmental stages

−*2.1prox1a* is a conserved sequence of 282 bp located upstream of the TSS (Fig. 1B; Fig. S1C). We recently reported (Kazenwadel et al., 2023) that this element is active in the developing tissue of the lymphatic valve at 5 dpf, and weakly in the rest of the facial lymphatics (Fig. 2A; Fig. S2A; Table 1). However, the functional characterisation of this enhancer and the timing of its expression in zebrafish are yet to be determined. Here, we uncovered that at 3 dpf the −*2.1prox1a* element is first active in the site of the future valve formation, preceding the *gata2a* onset of expression (Quillien et al., 2017), and its activity is ongoing until valve maturation at 7 dpf (Fig. 2A; Fig. S2A).

As the empty ZED vector induces low level of GFP expression in various tissues (Table 1; Fig. S2B) and it uses a *gata2a* minimal promoter (Bessa et al., 2009), we wanted to confirm the specificity of −*2.1prox1a*-driven expression. We re-cloned −*2.1prox1a* in a different plasmid, using an *e1b TATA* minimal promoter (Villefranc et al., 2007) which does not drive GFP expression without enhancer sequences (Fig. S2C; Table 1). We obtained the same enriched expression in the lymphatic valve and sparse expression in facial lymphatics (Fig. 2B,C; Table 1). We additionally investigated the enhancer activity in the other vascular valves present during embryonic development. Two lymphovenous valves (LVVs) have been described connecting the facial lymphatics to the veins (Meng et al., 2023; Shin et al., 2019). We detected weak activity of −*2.1prox1a* in the anterior LVV connecting the FCLV to the PHS, but none in the posterior LVV (Fig. S2D). No activity was detected in association with the developing atrio-ventricular and bulbo-ventricular cardiac valves (Fig. S2E). Whole-body imaging at 5 dpf revealed additional expression in a population of cells in the abdomen and extremely weak expression in few muscle cells (Fig. 2C), confirming the predominant activity of this enhancer in the lymphatic valve.

−*2.1prox1a* presents predicted binding sites for a variety of known lymphatic factors. At the 5′ it contains binding sites for Nfatc1 and Fox, previously reported to be conserved between zebrafish and mammals (Kazenwadel et al., 2023). In the mouse enhancer, a vertebrate-conserved Gata2 site (Fig. 2D; Tables S4,S5) has been confirmed in LECs using ChIP-seq that is functionally necessary for lymphatic vessel formation (Kazenwadel et al., 2023). However, this site is not present in zebrafish (Fig. 2D). The enhancer also includes binding sites for genes involved in vascular development such as forkhead box transcription factors (Niimi et al., 2017; Scallan et al., 2021), TCFs (Cha et al., 2016; Nicenboim et al., 2015), SNAI2 (Hultgren et al., 2020), retinoic acid receptors (Bowles et al., 2014; Marino et al., 2011) and ETS factors (Pham et al., 2007; De Val et al., 2008; Yoshimatsu et al., 2011) (Fig. 2D; Fig. S2F,G; Tables S4,S5). In order to test the functional relevance of the predicted TFs, we focused on the conserved Nfatc1 binding site. We injected embryos with the *e1b TATA* minimal promoter vector (Villefranc et al., 2007) containing either the wild-type (WT) −*2.1prox1a* sequence or one lacking the Nfatc1 site. We compared the level of activity in the valve at 5 dpf, and observed a 40% reduction of intensity in the mutated enhancer compared with the WT (Fig. 2E). No activity was observed in the scrambled-sequence controls (*n*=36). This suggests that Nfatc1 is indeed needed for the full activity of −*2.1prox1a*, but that even in its absence other factors can drive partial expression in the valve. Overall, these results show that despite some divergence, part of the upstream regulation of this enhancer is conserved between zebrafish and mammals.

### Accessible regions of open chromatin at the *prox1a* locus drive expression in the lymphatics

Using an evolutionary conservation approach, we have identified enhancers that are active in a subset of lymphatic endothelium, but no pan-lymphatic drivers. As non-conserved enhancers also exist, we complemented our conservation analysis using a chromatin accessibility approach, which can indicate the presence of an active enhancer in a tissue. To identify additional *prox1a* lymphatic enhancers we used a previously published single nuclei ATAC-seq (snATAC-seq) database of zebrafish ECs (*N*=3155) at 4 dpf, which included arterial ECs, venous ECs and LECs (Grimm et al., 2023). From control/WT sorted ECs, we selected all ECs and endocardium, and then subsetted and re-clustered the data (Fig. S3A). Cluster identity was determined using the gene accessibility score of marker genes of lymphatic and blood endothelium (Fig. S3A). Gene Ontology (GO) terms analysis revealed an enrichment for terms connected with lymphangiogenesis in the LEC-accessible regions, and enrichment for angiogenesis terms in the less accessible regions (Fig. S3B).

**Fig. 3.**
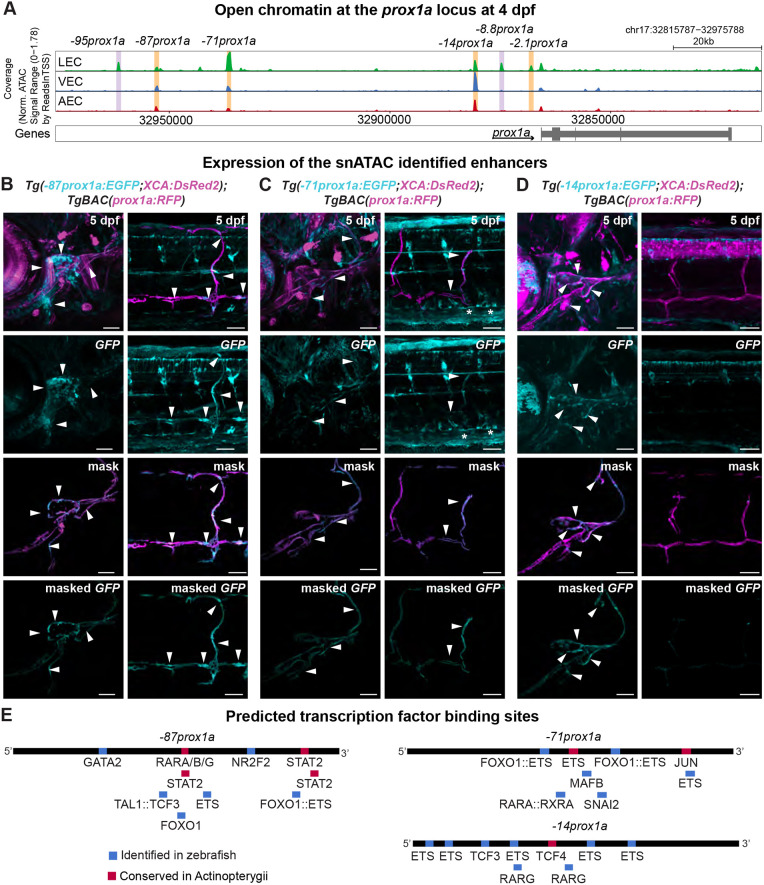
**snATAC-seq identifies four lymphatic *prox1a* enhancers.** (A) Chromatin state surrounding the *prox1a* locus in lymphatic endothelial cells (LECs), venous endothelial cells (VECs) and arterial endothelial cells (AECs) at 4 dpf, showing the region between 32,815,787−32,975,788 base pairs of chromosome (chr) 17. Orange, tested enhancers; purple, identified accessible chromatin sequences in LECs. (B) Confocal projections of the facial and trunk lymphatics labelled with *Tg(*−*87prox1a:EGFP; XCA:DsRed2)^uom122^* (cyan) and *Tg(prox1a:RFP)^nim5^* (magenta) at 5 dpf. Arrowheads show expression in the face and trunk lymphatics. (C) Confocal projections of the facial and trunk lymphatics labelled with *Tg(*−*71prox1a:EGFP; XCA:DsRed2)^uom121^* (cyan) and *Tg(prox1a:RFP)^nim5^* (magenta) at 5 dpf. Arrowheads show expression in the face and trunk lymphatics. Asterisks show expression in PCV. (D) Confocal projections of the facial and trunk lymphatics labelled with *Tg(*−*14prox1a:EGFP; XCA:DsRed2)^uom120^* (cyan) and *Tg(prox1a:RFP)^nim5^* (magenta) at 5 dpf. Arrowheads show expression in the facial lymphatics. (E) Predicted endothelial TF binding sites in −*87prox1a*. (*P*<1e-04), −*71prox1a* (*P*<1e-04) and −*14prox1a* (*P*<1e-04). Blue, binding sites identified in zebrafish; red, conserved binding sites within Actinopterygii. Scale bars: 50 μm.

We focused on the DNA region surrounding the *prox1a* locus and identified six regions of open chromatin in LECs (Fig. 3A). We established stable transgenic lines for three of them: −*87prox1a*, −*71prox1a* and −*14prox1a* (Table S2). All drove expression in the lymphatic endothelium (Table 1). Noticeably, −*2.1prox1a* was one of the elements retrieved by this analysis. We cloned the corresponding chromatin accessibility peak, named −*2.1prox1a_3*, which contained an additional 219 base pairs (bp) compared with −*2.1prox1a.* −*2.1prox1a_3* was able to induce expression in the lymphatic valve and residual expression in the rest of the facial lymphatic vessels (Fig. S3C,D)*.* This broader expression in the facial LECs is probably due to additional putative TF binding sites present in the sequence which regulate zebrafish lymphangiogenesis, such as Mafb (Arnold et al., 2022; Koltowska et al., 2015b). The +*15.2prox1a* enhancer does not appear to be accessible in either LECs or BECs at 4 dpf, which could be due to the dynamic activity of this element.

We further investigated whether the enhancers identified by snATAC-seq present sequence conservation within Actinopterygii. Microsynteny was tested in nine ray-finned fish species (Fig. S4A) confirming that the loss of *rps6kc1* upstream of *prox1a* we observed in zebrafish is a Otocephala-specific rearrangement and is not present in other Actinopterygii (Fig. S1A,C). The sequence conservation analysis revealed that −*71prox1a* is conserved across Actinopterygii, and so is −*14prox1a* although with a weaker signal. In contrast, −*87prox1a* could not be identified with sequence conservation in any of the considered acanthopterygian species (*Oreochromis niloticus*, *Acanthochromis polycanthus*, *Takifugu rubipes* and *Amphiprion percula*) (Fig. S4B). However, the presence of this enhancer in *Lepisosteus oculatus* suggests it was present in the ancestor of Actinopterygii, and the sequence has subsequently diverged or was lost in the acanthopterygian lineage. The reduced level of conservation of −*87prox1a* and −*14prox1a* also explains why only −*71prox1a* is marked as conserved in the UCSC Genome Browser Multiz Alignment (Fig. S3E). As expected, both −*2.1prox1a* and +*15.2prox1a* are conserved across Actinopterygii (Fig. S4B).

The −*87prox1a* element, positioned 87 kbp upstream of the TSS and marked as a primed enhancer at 48 hpf (Fig. S1C), drove reporter expression in the facial and trunk lymphatics at 5 dpf (Fig. 3B; Fig. S3F), as well as in the skin and a cell population in the trunk (Fig. S3F). The enhancer contains predicted TF binding sites for vascular regulators such as GATA2, FOX, ETS, NR2F2, TAL1::TCF3 (Cha et al., 2016; Frye et al., 2018; Kazenwadel et al., 2015; Lin et al., 2010; Nicenboim et al., 2015; Niimi et al., 2017; Pham et al., 2007; Scallan et al., 2021; Tang et al., 2006; De Val et al., 2008; Yoshimatsu et al., 2011), as well as actinopterygian-conserved binding sites for RARA (Bowles et al., 2014; Marino et al., 2011) (Fig. 3E; Fig. S4C; Tables S4,S5).

The −*71prox1a* element is situated 71 kbp upstream of the *prox1a* TSS and drove reporter expression in the trunk and the facial lymphatics at 5 dpf (Fig. 3C), as well as the PCV, skin and gallbladder (Fig. S3G). It contained predicted binding sites for factors involved in vascular development such as MAFB and ETS factors (Arnold et al., 2022; Koltowska et al., 2015b; Pham et al., 2007; De Val et al., 2008; Yoshimatsu et al., 2011) (Fig. 3E; Fig. S3C; Tables S4,S5). The enhancer also presents a binding site for RARA (Bowles et al., 2014; Marino et al., 2011), FOX (Niimi et al., 2017), SNAI2 (Hultgren et al., 2020) and JUN, which is part of the MAPK activation cascade downstream of VEGF signalling (reviewed by Guo et al., 2020) (Fig. 3E; Fig. S4D; Tables S4,S5).

Marked as a primed enhancer at 48 hpf, −*14prox1a* is situated 14 kbp upstream of *prox1a* (Fig. S1C). Within lymphatics, the enhancer activity is restricted to the face (Fig. 3D), but is also expressed in the skin (Fig. S3H). It contains predicted binding sites for vascular relevant factors, such as ETS factors (Pham et al., 2007; De Val et al., 2008; Yoshimatsu et al., 2011), FOX TF (Niimi et al., 2017), TCFs (Cha et al., 2016; Nicenboim et al., 2015) and RARG (Bowles et al., 2014; Marino et al., 2011) (Fig. 3E; Fig. S4E; Tables S4,S5).

In conclusion, we identified three additional lymphatic *prox1a* enhancers by means of chromatin state. Given that these elements show reduced or absent sequence conservation outside of Actinopterygii, this suggests evolutionary divergence in gene regulation – either through lineage-specific modification in *prox1a* regulation or through a change at the level of DNA sequence while retaining function.

### −*2.1prox1a_1* drives expression in the lymphatic valve

**Fig. 4.**
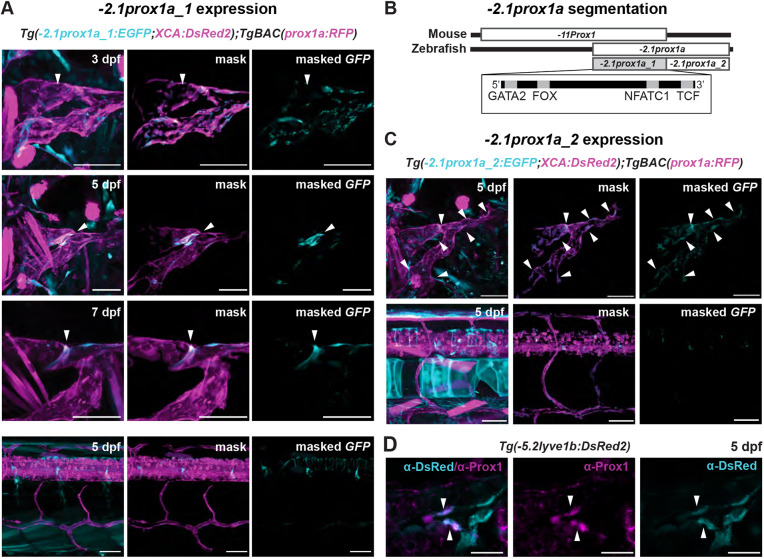
**−*2.1prox1a_1* is the core element driving valve expression.** (A) Confocal projections of the facial lymphatics labelled with *Tg(*−*2.1prox1a_1:EGFP; XCA:DsRed2)^uu5kk^* (cyan) and *Tg(prox1a:RFP)^nim5^* (magenta) at 3, 5 and 7 dpf, and lack of expression in the trunk lymphatics (bottom panel) at 5 dpf. Arrowheads show expression in the developing lymphatic valve. (B) Schematic of the −*2.1prox1a* zebrafish and the −*11Prox1* mouse enhancer. The identified sequence overlap between the two enhancers is referred to as −*2.1prox1a_1* and the zebrafish unique enhancer part is referred to as −*2.1prox1a_2*. The TF binding site location in −*2.1prox1a_1* is illustrated in the box. (C) Confocal projections of the facial and trunk lymphatics labelled with *Tg(*−*2.1prox1a_2:EGFP;XCA:DsRed2)^uu6kk^* (cyan) and *Tg(prox1a:RFP)^nim5^* (magenta) at 5 dpf. Arrowheads show expression in the facial lymphatics. (D) Confocal projections of the immunostaining against Prox1 (magenta) and *Tg(*−*5.2lyve1b:DsRed2)^nz101^* (cyan). Prox1 expression is detected in the valve leaflets at 5 dpf (arrowheads). Scale bars: 50 μm (A,C); 20 μm (D).

The −*2.1prox1a* element partially overlaps with that of a previously described −*11Prox1* murine lymphatic valve enhancer (Kazenwadel et al., 2023) in its 5′ portion (Fig. 4B). We hypothesised that this region might be sufficient to induce the valve-specific expression. The 200 bp fragment, called −*2.1prox1a_1*, was cloned in the ZED vector and tested *in vivo*. Expression driven by −*2.1prox1a_1* appeared at 3 dpf at the future valve position (Fig. 4A; Fig. S5A,B), which mirrors the observations for the full enhancer. The enhancer-driven valve expression was maintained at 5 dpf and 7 dpf (Fig. 4A), aligning with the endogenous Prox1 protein distribution at 5 dpf, which is especially concentrated in the leaflets of the developing valves (Fig. 4D). Similar to −*2.1prox1a*, −*2.1prox1a_1* remained inactive in the trunk lymphatics (Fig. 4A) and all venous tissues (Fig. 4A; Fig. S5D). Furthermore, we explored the potential role for the 3′ portion of −*2.1prox1a*, here called −*2.1prox1a_2*, as a complementary contributor to −*2.1prox1a_1* activity. Upon *in vivo* testing, this element showed subtle GFP expression in the facial, but not in the trunk, lymphatics (Fig. 4C; Fig. S5C), and no venous expression (Fig. S5D). This suggests that this segment of −*2.1prox1a* also plays a role in regulating *prox1a* expression.

In summary, −*2.1prox1a_1* serves as the core enhancer element of −*2.1prox1a*, sufficient to drive expression in the developing valve from the early stages to later developmental phases.

### −*2.1prox1a_1* is necessary for correct valve morphology and functionality

**Fig. 5.**
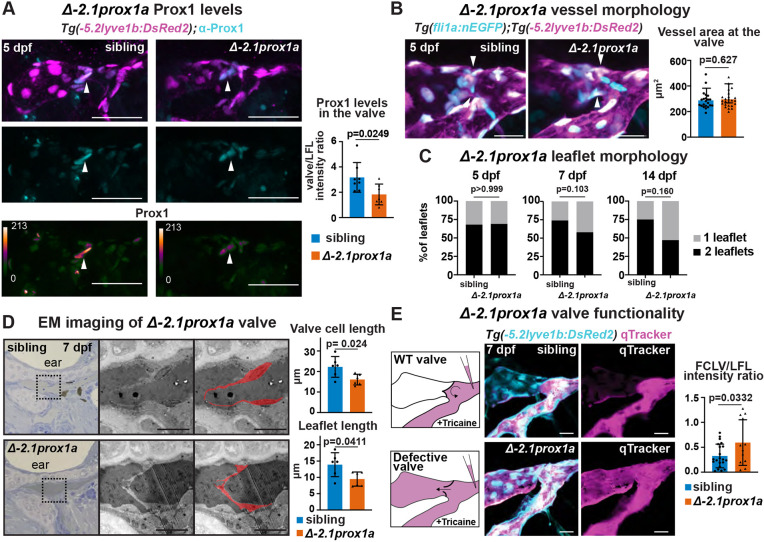
**−*2.1prox1a* is necessary for correct valve development and function.** (A) Top and middle: confocal projections of immunostaining against Prox1 (cyan) and *Tg(*−*5.2lyve1b:DsRed2)^nz101^* (magenta) in sibling and Δ−*2.1prox1a* mutant embryos at 5 dpf. Bottom: heatmap visualisation of Prox1 protein levels in sibling and Δ−*2.1prox1a* mutant embryos. Left: quantification of Prox1 protein levels in the valves of 5 dpf embryos. Mean±s.d. Sibling (*n*=8) versus mutants (*n*=7). *P*=0.0249 (unpaired two-tailed Student's *t*-test). Three technical replicates, biological replicates correspond to the number of data points per condition. (B) Right: confocal projections of *Tg(fli1:nEGFP)^y7^ (cyan)* and *Tg(*−*5.2lyve1b:DsRed2)^nz101^* (magenta) in sibling and Δ−*2.1prox1a* mutant embryos at 5 dpf. Left: quantification of vessel section at the valve (arrowheads) in Δ−*2.1prox1a* embryos at 5 dpf. Mean±s.d. Sibling (*n*=22) versus mutants (*n*=24). Not significant (ns) (*P*=0.627; two-tailed Mann–Whitney test). Three technical replicates, biological replicates correspond to the number of data points per condition. (C) Quantification of leaflet morphology in the valves of 5, 7 and 14 dpf sibling and Δ−*2.1prox1a* embryos. Mean±s.d. 5 dpf siblings (*n*=19) versus Δ−*2.1prox1a* (*n*=26), ns (*P*>0.999; Fisher's test). 7 dpf siblings (*n*=27) versus Δ−*2.1prox1a* (*n*=29), ns (*P*=0.103; Fisher's test). 14 dpf siblings (*n*=12) versus Δ−*2.1prox1a* (*n*=21), ns (*P*=0.160; Fisher's test). Three technical replicates, biological replicates correspond to the number of data points per condition. (D) Left: brightfield and TEM imaging of sibling (*n*=3) and Δ−*2.1prox1a* (*n*=3) valves at 7 dpf. The leaflets in the TEM images are highlighted in red. Right: quantification of cell length: mean±s.d. Siblings (*n*=6) versus Δ−*2.1prox1a* (*n*=6). *P*=0.024 (unpaired two-tailed Student's *t*-test). Quantification of leaflet length: mean±s.d. Siblings (*n*=6) versus Δ−*2.1prox1a* (*n*=6). *P*=0.0411 (unpaired two-tailed Student's *t*-test). Two technical replicates, biological replicates correspond to the number of data points per condition. (E) Left: schematic and confocal projections of *Tg(*−*5.2lyve1b:DsRed2)^nz101^ (cyan)* and Qtracker (magenta) in sibling and Δ−*2.1prox1a* mutant embryos at 7 dpf, visualising flow through the vessel. Right: quantification of Qtracker leakage through the valve in sibling and Δ−*2.1prox1a* embryos at 7 dpf. Mean±s.d. Siblings (*n*=21) versus mutants (*n*=12). *P*=0.0332 (unpaired two-tailed Student's *t*-test). Two technical replicates, biological replicates correspond to the number of data points per condition. Scale bars: 50 μm (A); 20 μm (B,E); 10 μm (D).

To test the necessity of these enhancers for the development of lymphatic vasculature, we concentrated on the core valve element −*2.1prox1a_1* and used CRISPR/Cas9 to generate the *en*−*2.1prox1a^uu12kk^* mutant line, hereafter referred to as Δ−*2.1prox1a*, carrying a 102 bp deletion covering the predicted NFATC1 binding site in the −*2.1prox1a_1* sequence. Homozygous mutants are viable, fertile and show normal body morphology. We focused on the valve area to characterise the phenotype. Immunostainings revealed reduced Prox1a protein levels within the valve of mutant Δ−*2.1prox1a* compared with siblings (Fig. 5A), suggesting the deletion of the enhancer negatively impacts the regulation of *prox1a*. We then investigated gross vessel morphology at the valve position, which showed high resemblance between Δ−*2.1prox1a* homozygous and sibling embryos (Fig. 5B; Fig. S6A-C). Similarly, the volume of the FCLV remained unaffected in Δ−*2.1prox1a* embryos (Fig. S6D). Using the average phenotype approach (Arnold et al., 2022) at 5 and 7 dpf, we further investigated the vessel morphology. No discernible differences were observed (Fig. S6E), further confirming that the facial lymphatic vessels form accurately in the mutants. Conversely, an analysis of gross valve morphology at 7 and 14 dpf revealed a noticeable trend of leaflet alteration in the mutants compared with the sibling (Fig. 5C). As 7 dpf is the stage in which the valve leaflets are completely formed (Shin et al., 2019), we further investigated the fine morphology of the valve using transmission electron microscopy (TEM). Imaging of three embryos per genotype revealed altered valve morphology in 7 dpf mutant embryos, with a reduction in length of both the cells composing the leaflet and the leaflets themselves (Fig. 5D). Roundness of the valve cell nuclei was unaffected (Fig. S6F).

A correctly formed valve should be closed under tricaine anaesthesia and therefore in an anaesthetised WT embryo the Qtracker injected in the lateral facial lymphatic (LFL) should not leak into the FCLV. However, when the valve is not correctly formed, and cannot close properly, the Qtracker can leak into the FCLV (Fig. 5E) (Shin et al., 2019). To assess whether the altered morphology could affect valve function, we performed Qtracker injections in the LFL in anaesthetised 7 dpf larvae and quantified leakage through the valve. Our findings demonstrated increased leakage in Δ−*2.1prox1a* mutants compared with siblings, confirming a functional impairment of the lymphatic valve in the absence of the enhancer (Fig. 5E; Fig. S6G). Collectively, these data show the necessity for −*2.1prox1a_1* for correct valve formation and proper expression of *prox1a*, highlighting the functional importance of *prox1a* enhancers for precise lymphatic development.

## DISCUSSION

**Fig. 6.**
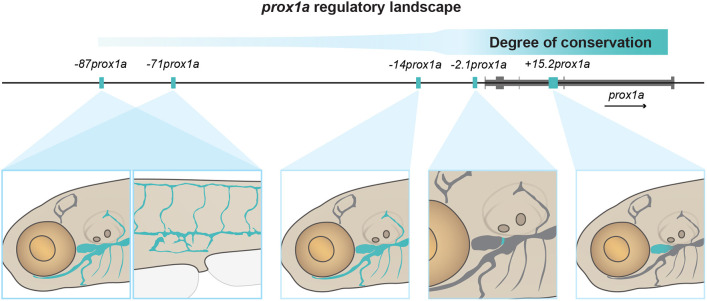
**Schematic representation of the lymphatic *prox1a* enhancers identified in this study.** The more distal enhancers, not conserved in mammals, drive expression in wide lymphatic domains. The proximal enhancers, conserved in mammals, show instead restricted activity in specific subsets of the lymphatic vasculature.

PROX1 is a key factor for LEC development (Koltowska et al., 2015a; Wigle and Oliver, 1999). Despite this, only one *cis*-regulatory element of *Prox1* has been identified so far (Kazenwadel et al., 2023). Here, we used a mixed approach, taking advantage of both evolutionary conservation and tissue-specific chromatin accessibility, to identify *prox1a* enhancers active in the lymphatic endothelium of zebrafish. Unexpectedly, we could identify five separate enhancers driving reporter expression in the lymphatic vasculature (Fig. 6). Two of the enhancers, −*2.1prox1a* and +*15.2prox1a*, show significantly enriched expression in the valve and the FCLV, respectively. These anatomically and functionally distinct subsets of the lymphatics are also regulated by different gene networks than the remaining lymphatics (Hußmann et al., 2023; Meng et al., 2023; Shin et al., 2019). Conversely, the area of activity of the three remaining enhancers is wider and overlaps to a larger extent. Specifically, both −*87prox1a* and −*71prox1a* drive expression in trunk and facial lymphatics*.* Enhancers with high redundancy in their expression patterns are defined as shadow enhancers (Hobert, 2010; Hong et al., 2008). Shadow enhancers are a common feature among developmental genes (Cannavò et al., 2016; Kvon et al., 2021). They are thought to serve as a mechanism aimed at ensuring robustness during the developmental process (Antosova et al., 2016; Kvon et al., 2021; Osterwalder et al., 2018). Various types of interactions – additive, superadditive, subadditive and repressive – among these enhancers have been shown to effectively fine-tune the regulation of target genes into specific patterns (Bothma et al., 2015; El-Sherif and Levine, 2016; Kvon et al., 2021; Lam et al., 2015). Moreover, studies indicate that shadow enhancers can form transcriptional hubs, where multiple enhancers concurrently interact with the gene promoter at the same time (Kvon et al., 2021). Given this intricate interplay, the presence of two enhancers, −*87prox1a* and −*71prox1a*, with extensively overlapping expression suggests a potential role for shadow enhancers in *prox1a* regulation. The complex regulatory logic at the *prox1a* locus highlights the necessity for tight regulation of *prox1a* activity to ensure correct lymphatic development.

Our conservation analysis revealed a high density of CNEs surrounding the *prox1a* locus. As CNEs can be considered putative enhancers, this suggests that *cis*-regulation plays an important role in controlling *prox1a* expression in more than just LECs. In fact, among ten tested CNEs, only two functional lymphatic enhancers were identified: −*2.1prox1a* and +*15.2prox1a*. In contrast, the three sequences identified by chromatin state, −*87prox1a*, −*71prox1a* and −*14prox1a,* presented varying conservation levels within Actinopterygii. Although distal enhancers have a tendency to be less conserved, the presence of other vertebrate-conserved CNEs spanning the 150 kbp upstream region of *prox1a* suggests that no major rearrangement has contributed to the loss of these enhancers in tetrapods. In addition, we observed conservation of microsynteny surrounding *prox1a* in tetrapods and actinopterygians, with the exception of an Otocephala-specific divergence. Such conservation in loci disposition also speaks against major genomic rearrangements in the region. Interestingly, the most conserved enhancers at the sequence level also have the most spatially restricted expression (the lymphatic valve and the FCLV), whereas the less conserved enhancer sequences with tetrapods display broader and overlapping expression patterns. Despite the obvious morphological differences, the molecular programme underlying LEC development in mammals and zebrafish presents strong similarities, such as the role of growth factor signalling and TFs such as PROX1, NFATC1 or GATA2 (Kazenwadel et al., 2015; Koltowska et al., 2015a; Norrmén et al., 2009; Secker and Harvey, 2021; Shin et al., 2019; Wigle and Oliver, 1999). Therefore, it is worth noticing that the majority of the binding sites for these conserved lymphatic regulators are found in the sequence-conserved enhancers. This suggests potential divergence in the molecular code upstream of −*87prox1a*, −*71prox1a* and −*14prox1a* between Actinopterygii and Tetrapoda, possibly involving different TFs. However, it is important to note that evolutionary conservation of functional enhancers does not necessarily require high levels of conservation at a sequence level, as long as key transcription factor motifs endure (Wong et al., 2020). As the nature of the enhancers can be complex, adopting complementary methods for their identification is crucial for a comprehensive understanding of a gene regulatory landscape.

As we just discussed, sequence conservation can serve as an indicator of enhancer presence; however, it does not guarantee complete functional conservation. In the case of −*11Prox1/*−*2.1prox1a*, the element drives expression in the lymphatic valve in both mouse and zebrafish. Both in mouse and zebrafish, *Prox1/prox1a* is a key player in lymphatic valve formation acting in concert with *Foxc1*, *Nfatc1* and *Gata2* (Bazigou et al., 2009; Norrmén et al., 2009; Sabine et al., 2012; Shin et al., 2019) and activating key downstream targets for valve development such as integrin alpha 9 (*Itga9*; Bazigou et al., 2009; Shin et al., 2019) and connexin 37 (*Gja4*; Sabine et al., 2012). The conserved cluster of binding sites for Foxc, Nfatc1 and Gata2 in the −*11Prox1/*−*2.1prox1a* enhancer strongly suggests this enhancer is part of the network. Despite this, the mice and zebrafish phenotypes for −*11Prox1/*−*2.1prox1a* mutants are profoundly different. The −*2.1prox1a* mutants are viable and fertile, and only show developmental defects in the lymphatic valve regarding the morphology of the leaflets. As mentioned above, key factors in the regulation of the leaflet extracellular matrix such as *itga9* (Bazigou et al., 2009) have been suggested to be regulated under the Prox1/Foxc/Nfatc1/Gata2 network in zebrafish lymphatic valve formation (Shin et al., 2019), which could explain the observed phenotype. Conversely, mouse −*11Prox1* mutants have more severe lymphatic defects and die perinatally (Kazenwadel et al., 2023). The deletion of −*11Prox1* is fully phenocopied by the deletion of the GATA2 binding site, which is also responsible for the transition of LECs to HECs in −*11Prox1* mutant mice (Kazenwadel et al., 2023). However, the putative GATA2 binding site is not conserved in zebrafish, which could again explain the different severity of the phenotype. The absence of this binding site is also the reason the hematopoietic phenotypes were not further explored in this study. The observed differences between −*11Prox1* and −*2.1prox1a* suggest functional divergence despite their common evolutionary origin, and highlights that functional conservation cannot be solely deduced from enhancer sequence data.

In conclusion, this study has demonstrated that *prox1a* in the lymphatic vasculature is regulated by a diverse group of distinct enhancers. We uncovered that the distal enhancers drive the expression in large regions of the lymphatic endothelium, whereas the proximal and sequence-conserved enhancers channel the expression to the functionally distinct sub-compartments of the developing lymphatic vascular network. This work represents a first step towards a full characterisation of the *cis*-regulatory landscape of *prox1a* and the understanding of the complex mechanisms regulating its lymphatic expression.

## MATERIALS AND METHODS

### Zebrafish

Zebrafish (*Danio rerio*) work was carried out with ethical approval from the Swedish Board of Agriculture (5.8.18-10590/2018 and 5.8.18-06282/2023) and in alignment with guidelines set by animal ethics committees at The University of Melbourne, ‘Zebrafish Breeding and Husbandry Ethics (Ethics ID 22235)’. The fish were maintained at the Genome Engineering Zebrafish National Facility (SciLifeLab, Uppsala, Sweden), the CIV (Centre for *In Vivo*, Uppsala University, Sweden) and the *Danio rerio* University of Melbourne facility (DrUM, Melbourne, Australia). Adults and embryos were housed according to standard procedures (Aleström et al., 2020). The previously published lines used in this study were *Tg(*−*5.2lyve1b:DsRed2)^nz101^* (Okuda et al., 2012), *TgBAC(prox1a:KalTA4-4xUAS-ADV. E1b:TagRFP)^nim5^*, referred to as *Tg(prox1a:RFP)^nim5^* in this study, (Dunworth et al., 2014; van Impel et al., 2014), *Tg(kdr-l:ras-cherry)^s916^* (Hogan et al., 2009), *Tg(fli1a:nEGFP)^y7^* (Lawson and Weinstein, 2002) and *Tg(*−*2.1prox1a:EGFP;XCA:DsRed2)^uu3kk^* (Kazenwadel et al., 2023).

The *Tg(*−*2.1prox1a_1:EGFP;XCA:DsRed2)^uu5kk^*, *Tg(*−*2.1prox1a_2:EGFP; XCA:DsRed2)^uu6kk^*, *Tg(*+*15.2prox1a:EGFP;XCA:DsRed2)^uu7kk^*, *Tg(*−*2.1prox1a_3:EGFP; XCA:DsRed2)^uom119^*, *Tg(*−*71prox1a:EGFP; XCA:DsRed2)^uom121^*, *Tg(*−*87prox1a:EGFP; XCA:DsRed2)^uom122^*, *Tg(*−*14prox1a:EGFP; XCA:DsRed2)^uom120^*, *Tg(*−*2.1prox1a:basEGFP;ACry:GFP)^uu10kk^*, *en.*−*2.1prox1a^uu12kk^* and *Tg(gata-i4:GFP)^uu11kk^*, *Tg(*+*15.2prox1a:basEGFP;ACry:GFP)^uu13kk^*, *Tg(*−*87prox1a:basEGFP;ACry:GFP)^uu14kk^*, *Tg(*−*71prox1a:basEGFP;ACry:GFP)^uu15kk^*, *Tg(*−*14prox1a:basEGFP;ACry:GFP)^uu16kk^* lines were generated for this study.

### *In-silico* predictions

To identify sequence-conserved enhancers, the DNA regions between the two loci neighbouring *Prox1*, *prox1a* or *prox3* were downloaded from ENSEMBL, together with annotations. For *prox3*, homologs were identified using BLAST of zebrafish *prox3* and confirmed by local synteny. The sequences were oriented in the direction of the transcription. The species, assemblies and regions used are listed in Table S1. CNEs were identified using mVISTA non-coding DNA conservation analysis (Dubchak et al., 2000; Frazer et al., 2004; Mayor et al., 2000). The alignment was performed using the LAGAN program (Brudno et al., 2003).

To determine the zebrafish-specific binding sites in the identified *prox1a* enhancers, we conducted a motif discovery analysis of zebrafish enhancer sequences using FIMO in the MEME Suite (Grant et al., 2011). Our motif prediction was constrained to a search for motifs of size 7, with a *P*-value cut-off of 1e-04. A comprehensive list of predicted motifs is shown in Table S6.

To identify evolutionarily conserved motifs and binding sites within the specified enhancers, we retrieved the identified conservation peaks and subjected them to analysis using the MEME Suite (Bailey and Elkan, 1994) and TOMTOM (Gupta et al., 2007). A list of the conserved motifs identified can be found in Table S4. To validate the predicted conserved binding sites, we compared the results of this analysis with the outcomes of the FIMO motif discovery analysis, using a *P*-value cut-off of 1e-02. The decision to use a higher *P*-value stems from the conservation of these sequences and aims to capture an informative overview of the potential binding sites. Binding sites for known endothelial factors that appear in both analyses have been reported, and a list can be found in Table S5.

### snATAC-seq data processing and analysis

For snATAC-seq data analysis, we included only the 4 dpf WT cells from the publicly available dataset (Grimm et al., 2023). The analysis was performed and data were processed as previously described (Grimm et al., 2023). Briefly, the LEC cluster was defined by combined high accessibility at the *prox1a*, *cdh6* and *lyve1b* loci. The venous endothelial cells cluster by combined high accessibility at the *cdh5*, *kdrl* and *stab2* or *lyve1b* loci, and arterial endothelial cells by combined high accessibility at the *cdh*5, *kdrl*, *flt1* and *dll4* loci but low accessibility at *lybe1b*. Mural lymphatic endothelial cells (MuLECs) were identified based on the high accessibility at *osr2* in addition to the standard LEC loci but low accessibility at *cdh6*, and the endocardium by the accessibility at the *cdh5* and *hand2* loci.

All GO analyses were performed using Panther.db (Thomas et al., 2006) (Biological Process Complete). The complete list of the predicted GO terms is shown in Table S7.

### Cloning and transgenesis

The conserved elements of interest (Tables S2 and S3) were cloned into the ZED vector as previously described (Bessa et al., 2009) (Addgene plasmids 218205, 218206, 218207 and 218208). The *Tg(*−*2.1prox1a:basEGFP;ACry:GFP)^uu10kk^* line (Addgene plasmid 218209) was generated by cloning the −*2.1prox1a* sequence in a p5E-MCS vector (Quillien et al., 2017) (Addgene vector 26029) using In-Fusion cloning (Takara Bio, primers are listed in Table S3) and then inserted into a pDestTol2ACryGFP backbone (Berger and Currie, 2013) (Addgene vector 64022), with a pENTRbasEGFP (Villefranc et al., 2007) (Addgene plasmid 22453) and a p3E-polyA (Tol2kit v1.2 #302) vector using the Gateway cloning method (Invitrogen). ATAC-identified enhancers were inserted into the ZED vector by In-Fusion cloning (#638910, In-Fusion HD Cloning Plus Kits, Takara Bio) using BspEI and BmgBI cutting sites for linearisation. Primers used are listed in Table S3. Vectors used to generate the *Tg(*+*15.2prox1a:basEGFP;ACry:GFP)^uu13kk^* (Addgene plasmid 218210), *Tg(*−*87prox1a:basEGFP;ACry:GFP)^uu14kk^* (Addgene plasmid 218213), *Tg(*−*71prox1a:basEGFP;ACry:GFP)^uu15kk^* (Addgene plasmid 218212), *Tg(*−*14prox1a:basEGFP;ACry:GFP)^uu16kk^* (Addgene plasmid 218211) stable lines, as well as the transient −*2.1prox1a:basEGFP;ACry:GFP* with WT, NFATC1-mutated (Addgene plasmid 218215) and scrambled-sequence (Addgene plasmid 218216) transient transgenic embryos (Fig. 2E) were ordered from GenScript using the same backbone used for −*2.1prox1a:basEGFP;ACry:GFP* (Addgene plasmid 218214). To generate transgenic lines, 1 μl of construct at 20 ng/μl and *tol2* transposase mRNA at 100 ng/μl were injected into the one-cell stage WT zebrafish embryos. F0 embryos were screened for reporter expression and F1 embryos were screened using confocal microscopy for GFP expression in the lymphatic structures. The numbers of F0 founders screened for each tested CRE are listed in Table S2. Bleed-through in the GFP channel was excluded by imaging the embryos that were negative for *TgBAC(prox1a: RFP)^nim5^* (Fig. S5E)*.* F1 fish with lymphatic GFP expression were used to establish the stable lines. The *Tg(gata2a-i4-1.1kb:GFP)^uu11kk^* was created by injecting the construct (Dobrzycki et al, 2020) as previously described. For embryos imaged at F0, injections were performed as described for stable transgenic lines. The plasmids produced in this study have been deposited in Addgene.

### Imaging and image processing

For the conserved enhancer reporter and mutant lines, transgenic embryos were anaesthetised with tricaine, mounted in 1% low-melting agarose, and face or trunk was imaged using a Leica TCS SP8 DLS microscope with a Fluotar VISR 25× water objective (objective number: 11506375). For enhancers identified by snATAC-seq, transgenic embryos were anaesthetised with tricaine, mounted in 0.5% low-melting agarose and imaging was conducted at the Centre for Advanced Histology and Microscopy (Peter MacCallum Cancer Centre, Melbourne, Australia). Live samples were imaged using a Zeiss LSM 780 FCS confocal microscope. Images were processed using ImageJ software (v. 2.9.0). Immunostained embryos were mounted in clearing solution Omnipaque (350 mg/l concentration per 1 ml iohexol, GE Healthcare) and imaged using the Leica TCS SP8 DLS microscope as described above. All representative images are maximum intensity projections of the *z*-stack generated using ImageJ (v. 2.9.0). The skin signal in the GFP channel was manually removed to allow the visualisation of the vascular tissues underneath.

### Mutant line generation

Mutants were generated using CRISPR/Cas9 as described previously (Carrington et al., 2015; Varshney et al., 2016). The guides were designed to flank the target enhancer sequences. Zebrafish embryos were injected at the one-cell stage with 70-140 ng/μl of each gRNA and 200 ng/μl Cas9 mRNA. These guides are listed in Table S8. Fish were genotyped by PCR, followed by gel electrophoresis, and the deletion was confirmed by Sanger sequencing (Eurofins). F1 embryos with identical mutations were used to establish the stable mutant line.

### Genotyping

The Δ−*2.1prox1a* embryos were genotyped by PCR, followed by gel electrophoresis. PCR was performed as previously described for FLA genotyping (Carrington et al., 2015) using the primers listed in Table S3. Fluorescent primers were omitted from the reaction. *mafba* and *mafbb* mutant embryos were genotyped as previously described (Arnold et al., 2022).

### Immunostaining

Immunostaining was performed as previously described (Le Guen et al., 2014; Shin et al., 2016), with the addition of a 45 min at room temperature digestion step in proteinase K (PK) as described previously (Koltowska et al., 2015b). The primary antibodies used were anti-Prox1 rabbit (AngioBio, #11-002P, 1:100, Lot: GR3247830-10) (Koltowska et al., 2015a) and anti-mCherry chicken (AvesLabs, #MCHERRY-0020, 1:100, Lot: MC87977980). For the anti-mCherry primary antibody, we verified that the observed signal recapitulated the endogenous transgenic line expression. The secondary antibodies used were anti-rabbit IgG HRP (Cell Signaling Technology, #7076, 1:1000, Lot: 28) and anti-chicken 488 (Jackson ImmunoResearch, #703-545-155, 1:200, Lot: 158347). Signal amplification was performed using the TSA™ Plus Cyanine 3 System (Perkin Elmer, #NEL744001KT), with a development time of 3 h. Imaging was performed as described above.

### Electron microscopy imaging

For TEM, Δ−*2.1prox1a* embryos were fixed in 2.5% Glutaraldehyde (Ted Pella)+1% Paraformaldehyde (Merck) in 0.1 M Phosphate buffer (PB; pH 7.4), then embedded in 8% agar and a 300 μm sagittal section was cut on a Microm M 650V vibratome (Thermo Fisher Scientific) and stored at 4°C until further processing. The tails were then used for genotyping. Samples were rinsed with 0.1 M PB for 10 min before 1 h incubation in 1% osmium tetroxide (TAAB) in 0.1 M PB. After rinsing in 0.1 M PB, samples were dehydrated using increasing concentrations of ethanol (50%, 70%, 95% and 99.9%) for 10 min each step, followed by 5 min incubation in propylene oxide (TAAB). The samples were then placed in a mixture of Epon resin (Ted Pella) and propylene oxide (1:1) for 1 h, followed by 100% resin and left overnight. Subsequently, samples were embedded in capsules in newly prepared Epon resin and left for 1-2 h, and then polymerized at 60°C for 48 h.

Semi-thin sections were cut, stained with Toluidine Blue and examined using light-microscopy to identify the area of interest. Ultrathin sections (60-70 nm) were cut using an EM UC7 Ultramicrotome (Leica) and placed on a grid. The sections were subsequently contrasted with 5% uranyl acetate and Reynold's lead citrate and visualised with Tecnai™ G2 Spirit BioTwin transmission electron microscope (Thermo Fisher Scientific/FEI) at 80 kV with an ORIUS SC200 CCD camera and Gatan Digital Micrograph software (both from Gatan).

### Qtracker injections

Microangiography was performed following a previously published protocol (Arnold et al., 2022; Shin et al., 2019). Embryos were anaesthetised with Tricane and injected with 1 nl of Qtracker™ 655 Vascular labels (Thermo Fisher Scientific) at 7 dpf in the LFL for valve leakage experiments and imaged on a Leica TCS SP8 DLS microscope at ∼5 min post-injection.

### Image quantification

Intensity of +*15.2prox1* activity in WT and *mafba/b* mutant embryos was calculated in Imaris v9.3.0. Intensity of signal in the FCLV was calculated by manually segmenting the FCLV and extracting the intensity measurement. Intensity was normalised using the intensity signal in the *prox1a*-postive ganglia over the ear.

The mean intensity of GFP signal in the valve of embryos injected with WT and binding site-mutated −*2.1prox1a* embryos was calculated in Imaris v9.3.0. The GFP^+^ valve cells were isolated using a surface detail of 0.454 and a fixed thresholding value of 14. As no GFP^+^ cells were observed in the scrambled controls, the intensity could not be calculated in these embryos.

The relative levels of Prox1 protein in the valve were calculated as the ratio between the representative valve and LFL nucleus. Briefly, the chosen nuclei were masked in Imaris v9.3.0 as described above. The average intensity was calculated and used to compile the ratio. Nuclei with a maximum intensity of 255 were excluded from the calculations.

Vessel sections were calculated in ImageJ (v. 2.9.0) using a custom-made script, available at https://github.com/virpa81/Vessel-section-calculation. The *z*-stack was rotated along its main axis to ensure that the vessel section was perpendicular to the cutting planes. The stack was then re-sliced along the *yz*-plane, and the valve position was selected. Thresholding was used to mask off the vessel area, which was subsequently measured.

The FCLV volume was calculated in Imaris v9.3.0, by manually creating a surface spanning the 400 pixels of FCLV past the valve position and calculating its volume. To generate average phenotypes, images were acquired and processed as previously described (Arnold et al., 2022).

Valve leaflet morphology was scored in Imaris v9.3.0, looking at the projections of the stack in *x*, *y* and *z*, and scoring whether one or two leaflets were visible in any of them. To calculate the valve cell length in the TEM images, the cells composing the leaflets were traced manually and used to create a mask in ImageJ. The mask was then skeletonised and secondary branches were removed to obtain the length of the cell. Nuclei roundness was calculated in ImageJ using the ‘Round’ image descriptor. Leaflet length was calculated by tracing the internal edge of the leaflet in ImageJ and measuring.

Valve leakage in the injected embryos was quantified in ImageJ (v2.9.0). Intensity of the Qtracker signal was calculated in a representative slice by tracing a region of interest encompassing the FCLV or the LFL. Leakage was quantified as the ratio between these two measures.

### Statistical analysis

For all analysis, data were collected across at least three technical replicates, except for the TEM imaged embryos, the 3 dpf Δ−*2.1prox1a* embryos and the Qtracker injections, which were collected from two technical replicates. The number of biological replicates for each experiment is indicated in the figure legends.

Embryos presenting clear sign of altered general development were excluded from the analysis. *n* size was set as 10 per group, unless technical limitations prevented it. For injections of WT, mutated and scrambled vectors in Fig. 2E, injections were randomised by injecting different constructs into the same clutch. Quantifications of mutant embryos were performed before the assignment of the genotype, to ensure that experimenters were unaware of the conditions. The normality of all numerical datasets was tested with a Shapiro-Wilk test. For pair-wise comparison, an unpaired two-tailed Student's *t*-test was run on normally distributed data, while a Mann–Whitney test was run if normality was not confirmed. For multiple comparison, an Anova or Kruskal–Wallis test was used, depending on normality of the distribution. For frequency data, Fisher's exact test was used.

## Supplementary Material



10.1242/develop.202525_sup1 Supplementary information

Table S1. mVista analysis sequences

Table S2. Tested *prox1a* putative CREs

Table S3. Primers and gRNAs

Table S4. Conserved motifs detected in evolutionary conserved enhancers

Table S5. Predicted TFs binding sites in *prox1a* enhancers

Table S6. Complete list motifs based on zebrafish (p-val < 1e-04)

Table S7. GO terms enrichment in more and less accessible sequences in LECs

Table S8. gRNAs used

Table S5A. Predicted TFs binding sites in *prox1a* enhancers

Table S6A. Complete list motifs based on zebrafish (p-val < 1e-04)

Table S7A. GO terms enrichment in more and less accessible sequences in LECs

## Data Availability

Custom script has been deposited in GitHub (https://github.com/virpa81/Vessel-section-calculation).
