# Supplementary material for: Multiple cis-regulatory elements control prox1a expression in distinct lymphatic vascular beds
Source: Development. 2024 May 9;151(9):dev202525. doi: 10.1242/dev.202525 (PMC11128278; doi:10.1242/dev.202525)
Supplement: Supplementary information [file develop-151-202525-s1.pdf]

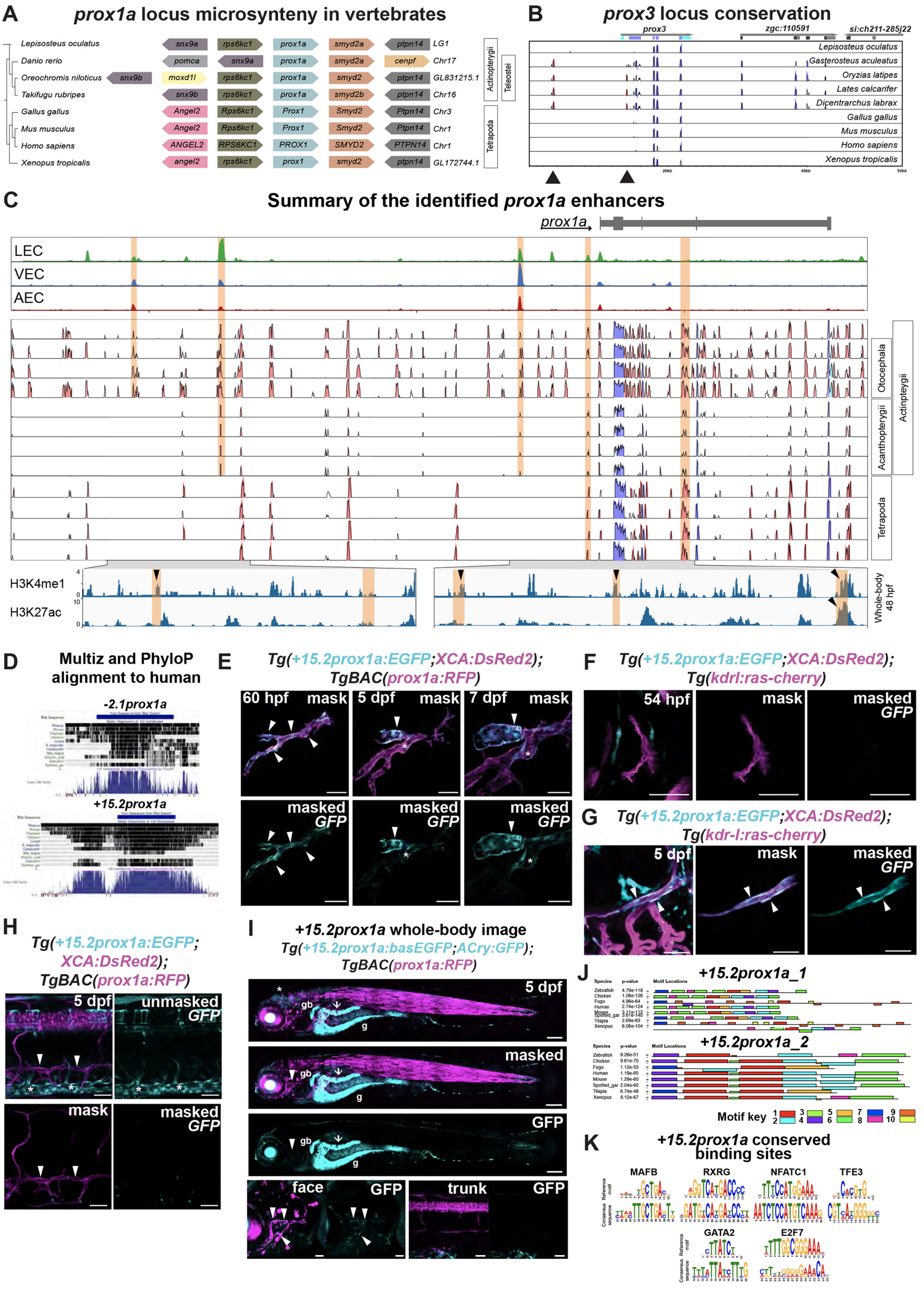

Development • Supplementary information

Fig. S1. CREs identification and additional *+15.2prox1a* enhancer characterisation (A) Microsynteny surrounding the vertebrate *Prox1/prox1a* locus. Downstream of *Prox1/prox1a*, *Smyd2* is found in all analysed sequences. Upstream of *Prox1/prox1a, Rps6ck1* is found in all species, except zebrafish. The annotations used are listed in Table S1.

(B) Conservation analysis of the 54 kbp region surrounding the *prox3* locus comparted to nine vertebrate species. Results are shown compared to zebrafish. Blue peaks: Exons; red peaks: Region of conserved non-coding DNA; black arrowheads: Conserved peaks.

(C) Schematics showing the identified *prox1a* enhancers, the snATAC signature in LEC and VEC, the conservation across Osteichthyes and the H3K4me1 and H3K27ac signature from whole embryos at 48 hpf, indicating primed and active enhancers. Orange: position of the identified enhancers. Arrowheads: H3K4me1 and H3K27ac signals in correspondence to the *prox1a* enhancers.

(D) Multiz alignment and PhyloP tracks from the UCSC Genome browser showing conservation across vertebrates of *+15.2prox1a* and *-2.1prox1a*. Conservation is shown compared to human.

(E) Masked confocal projections of the image in Fig. 1C. Arrowheads: expression in the facial LECs (60 hpf) and FCLV (5 and 7 dpf). Asterisks: Expression in facial lymphatic endothelium.

(F) Confocal projections of VA-L labelled with *Tg(+15.2prox1a:EGFP;XCA:DsRed2)^{uu7kk}* (cyan) and *Tg(prox1a:RFP)^{nim5}* (magenta) at 54 hpf, showing no lymphatic expression.

(G) Confocal projections of the primary head sinus labelled with *Tg(+15.2prox1a_1:EGFP;XCA:DsRed2)^{uu7kk}* at 5 dpf, showing *+15.2prox1a* driven venous expression (arrowhead).

(H) Confocal projections of trunk lymphatics labelled with *Tg(+15.2prox1a:EGFP; XCA:DsRed2)^{uu7kk}* (cyan) and *Tg(prox1a:RFP)^{nim5}* (magenta) at 5 dpf. Arrowheads: lymphatic vessels. Asterisks: posterior cardinal vein.

(I) Embryo labelled with *Tg(+15.2prox1a:basEGFP;ACry:GFP)^{uu13kk}* (cyan) and *Tg(prox1a:RFP)^{nim5}* (magenta). Arrowhead: expression in the FCLV and facial lymphatics. Asterisks: expression in the skin. Arrow: expression in the pancreas. g: expression in the gut. gb: expression in the gallbladder. Scale bar: 200 µm.

(J) Conserved motif structure of the identified lymphatic *+15.2prox1a* enhancer. Motif sequences are listed in Table S4.

(K) Predicted conserved binding site for *+15.2prox1a* in vertebrates. Motif locations are listed in Table S4.

Scales bars represent 50µm unless otherwise stated.

Development • Supplementary information

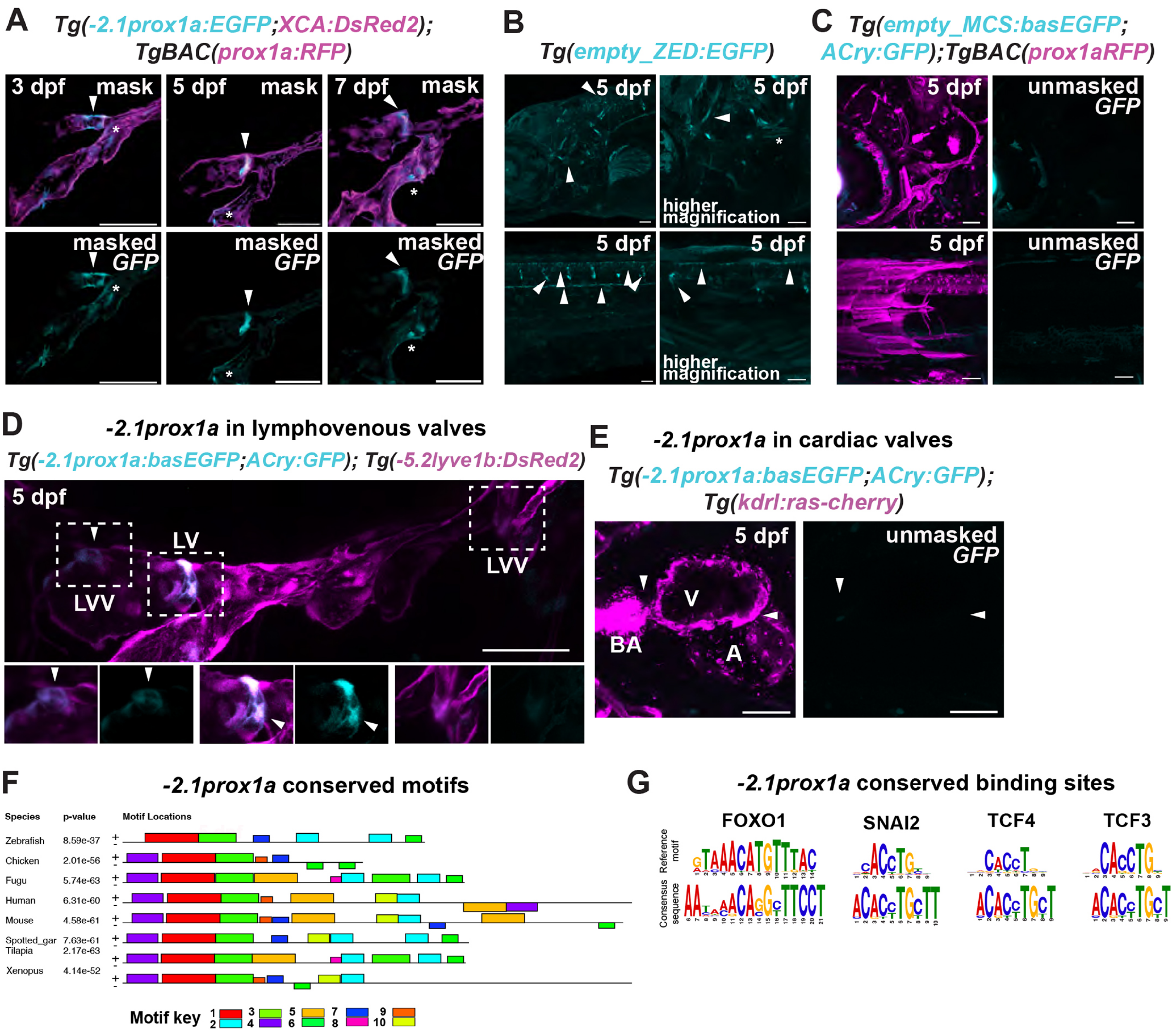

Fig. S2. Additional -*2.1prox1a* enhancer characterisation

(A) Masked confocal projections of the image in Fig. 2A. Arrowheads: Expression in the developing lymphatic valve. Asterisks: Expression in the facial lymphatic endothelium.

(B) Confocal projections of facial (top) and trunk (bottom) lymphatics at 5 dpf labelled with the empty ZED vector as a control. Arrowheads: Neuronal structures.

(C) Confocal projections of facial and trunk lymphatics at ˜5 dpf labelled with *Tg(prox1a:RFP)*^{nim5} (magenta) and injected with *empty_MSC:basEGFP;ACry:GFP* plasmid to create a transient control.

(D) Confocal projections of the LVVs at 5 dpf labelled with *Tg(-2.1prox1a: basEGFP;ACry:GFP)*^{uu10kk} (cyan) and *Tg(-5.2lyve1b:DsRed2)*^{nz101} (magenta). Boxes: LVVs and LV position. Arrowheads: expression in the LVV and LV.

(E) Confocal projections of the heart at 5 dpf labelled with *Tg(-2.1prox1a: basEGFP; ACry:GFP)*^{uu10kk} (cyan) and *Tg(kdr-l:ras-cherry)*^{s916} (magenta). A: atrium. V: ventricle. BA: bulbus arteriosus. Arrowheads: cardiac valves position.

(F) Conserved motif structure of the identified lymphatic -2.*1prox1a* enhancer. The motif sequences are listed in Table S4.

(G) Predicted conserved binding site for -*2.1prox1a* in vertebrates. Motif locations are listed in Table S4.

Scales bars represent 50µm.

Development • Supplementary information

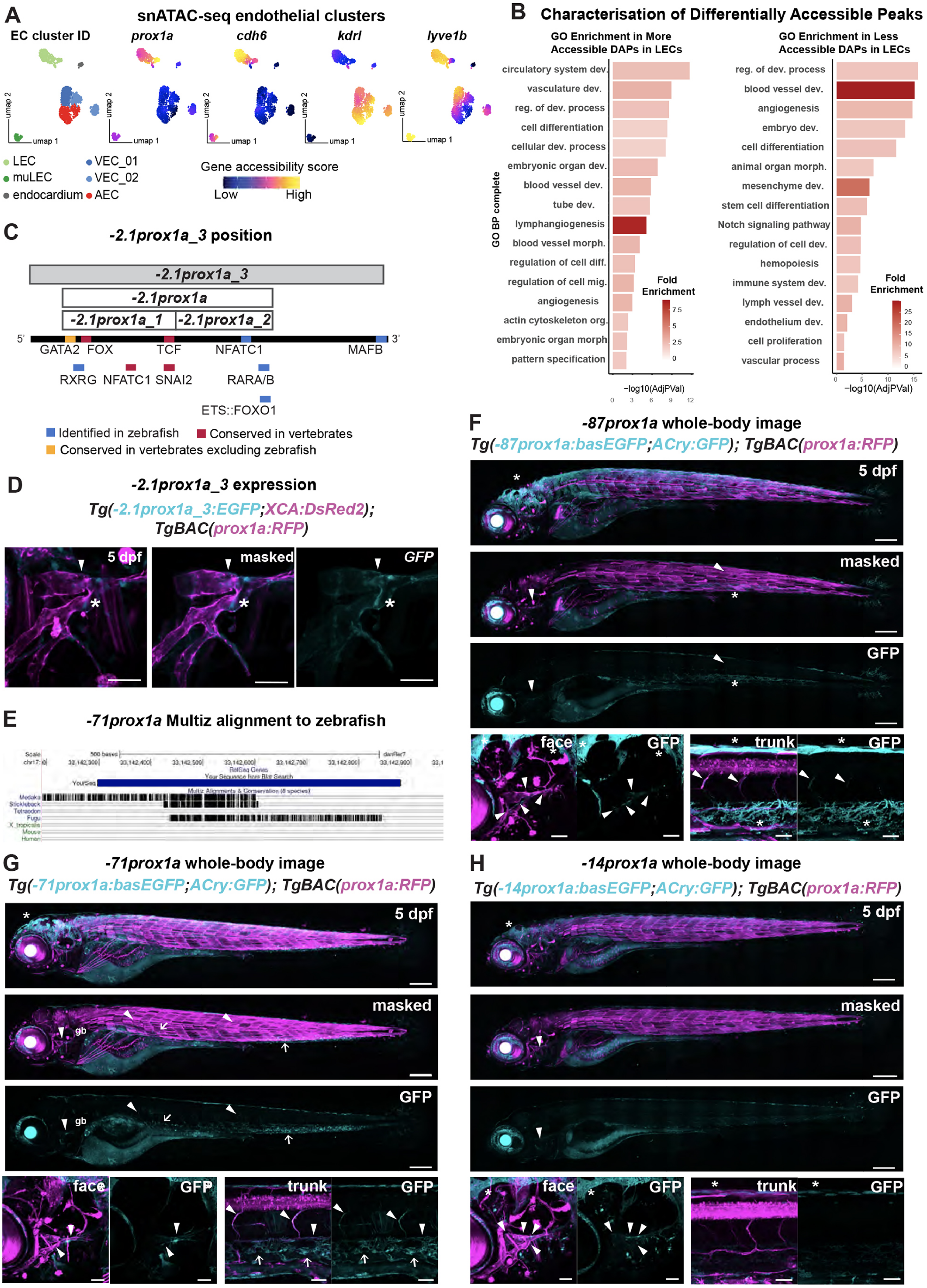
A
snATAC-seq endothelial clusters
EC cluster ID
prox1a
cdh6
kdrl
lyve1b
umap 1
umap 2
LEC
muLEC
endocardium
VEC_01
VEC_02
AEC
Gene accessibility score
Low
High
B
Characterisation of Differentially Accessible Peaks
GO Enrichment in More Accessible DAPs in LECs
GO Enrichment in Less Accessible DAPs in LECs
GO BP complete
circulatory system dev.
vasculature dev.
reg. of dev. process
cell differentiation
cellular dev. process
embryonic organ dev.
blood vessel dev.
tube dev.
lymphangiogenesis
blood vessel morph.
regulation of cell diff.
regulation of cell mig.
angiogenesis
actin cytoskeleton org.
embryonic organ morph
pattern specification
Fold Enrichment
−log10(AdjPVal)
reg. of dev. process
blood vessel dev.
angiogenesis
embryo dev.
cell differentiation
animal organ morph.
mesenchyme dev.
stem cell differentiation
Notch signaling pathway
regulation of cell dev.
hemopoiesis
immune system dev.
lymph vessel dev.
endothelium dev.
cell proliferation
vascular process
C
-2.1prox1a_3 position
-2.1prox1a_3
-2.1prox1a
-2.1prox1a_1
-2.1prox1a_2
5'
3'
GATA2
FOX
TCF
NFATC1
MAFB
RXRG
NFATC1
SNAI2
RARA/B
ETS::FOXO1
Identified in zebrafish
Conserved in vertebrates
Conserved in vertebrates excluding zebrafish
D
-2.1prox1a_3 expression
Tg(-2.1prox1a_3:EGFP;XCA:DsRed2); TgBAC(prox1a:RFP)
5 dpf
masked
GFP
E
-71prox1a Multiz alignment to zebrafish
F
-87prox1a whole-body image
Tg(-87prox1a:basEGFP;ACry:GFP); TgBAC(prox1a:RFP)
5 dpf
masked
GFP
face
GFP
trunk
GFP
G
-71prox1a whole-body image
Tg(-71prox1a:basEGFP;ACry:GFP); TgBAC(prox1a:RFP)
5 dpf
masked
GFP
gb
face
GFP
trunk
GFP
H
-14prox1a whole-body image
Tg(-14prox1a:basEGFP;ACry:GFP); TgBAC(prox1a:RFP)
5 dpf
masked
GFP
face
GFP
trunk
GFP

Fig. S3. snATAC-seq validation and additional ATAC-identified enhancers characterisation

(A) Clusters of endothelial cells (ECs) and endocardium at 4 dpf with umaps showing the chromatin state at known lymphatics and venous loci. High accessibility at *prox1a* and *cdh6* marks the lymphatic endothelial cell (LECs) cluster. High *kdrl* accessibility marks the venous endothelial cells (VECs) and arterial endothelial cell (AECs) cluster, and high *lyve1b* accessibility marks LECs and VECs. muLECs: Mural lymphatic endothelial cells.

(B) GO enrichment of developmentally relevant terms in differentially accessible peaks in LECs.

(C) Schematic representation of the *-2.1prox1a* enhancer indicating the overlap between the *-2.1prox1a_3* sequence from the identified peak using the ATAC-sequencing, *-2.1prox1a* identified using the conservation method (Fig. 1A) and the segmentation into *-2.1prox1a*_1 and *-2.1prox1a*_2 (Fig. 4B). Predicted binding for lymphatic-associated transcription factors is indicated below the enhancer fragments. Blue: Binding sites identified in zebrafish (p-val < 1e- 04). Red: Conserved binding sites within vertebrates. Yellow: Binding sites conserved in vertebrates but absent in zebrafish.

(D) Confocal projections of facial lymphatics labelled with *Tg(-2.1prox1a_3:EGFP; XCA:DsRed2)*^{uom119} (cyan) and *Tg(prox1a:RFP)*^{nim5} (magenta) at 5 dpf. Arrowheads: Expression in the developing lymphatic valve. Asterisks: expression in the facial lymphatic endothelium. Scale bars: 50µm.

(E) UCSC Genome browser Multiz alignment of the *-71prox1a* enhancer in zebrafish, showing conservation in Actinopterygii but not tetrapods.

(F-H) Tile-scan, face and trunk confocal projection of whole F1 embryo at 5dpf. In second and third panels, skin signal has been removed manually. Tile-scan scalebar = 200µm, zoom-in panels scalebar: 50µm.

(F) Embryo labelled with *Tg(-87prox1a:basEGFP;ACry:GFP)*^{uu14kk} (cyan) and *Tg(prox1a:RFP)*^{nim5} (magenta). Arrowhead: expression in the facial lymphatics. Asterisks: expression in the skin and connective tissue.

(G) Embryo labelled with *Tg(-71prox1a:basEGFP;ACry:GFP)*^{uu15kk} (cyan) and *Tg(prox1a:RFP)*^{nim5} (magenta). Arrowhead: expression in the lymphatics. Asterisks: expression in the skin. Arrows: expression in the PCV. gb: expression in the gallbladder.

(H) Embryo labelled with *Tg(-14prox1a:basEGFP;ACry:GFP)*^{uu16kk} (cyan) and *Tg(prox1a:RFP)*^{nim5} (magenta). Arrowhead: expression in the facial lymphatics. Asterisks: expression in the skin.

Development • Supplementary information

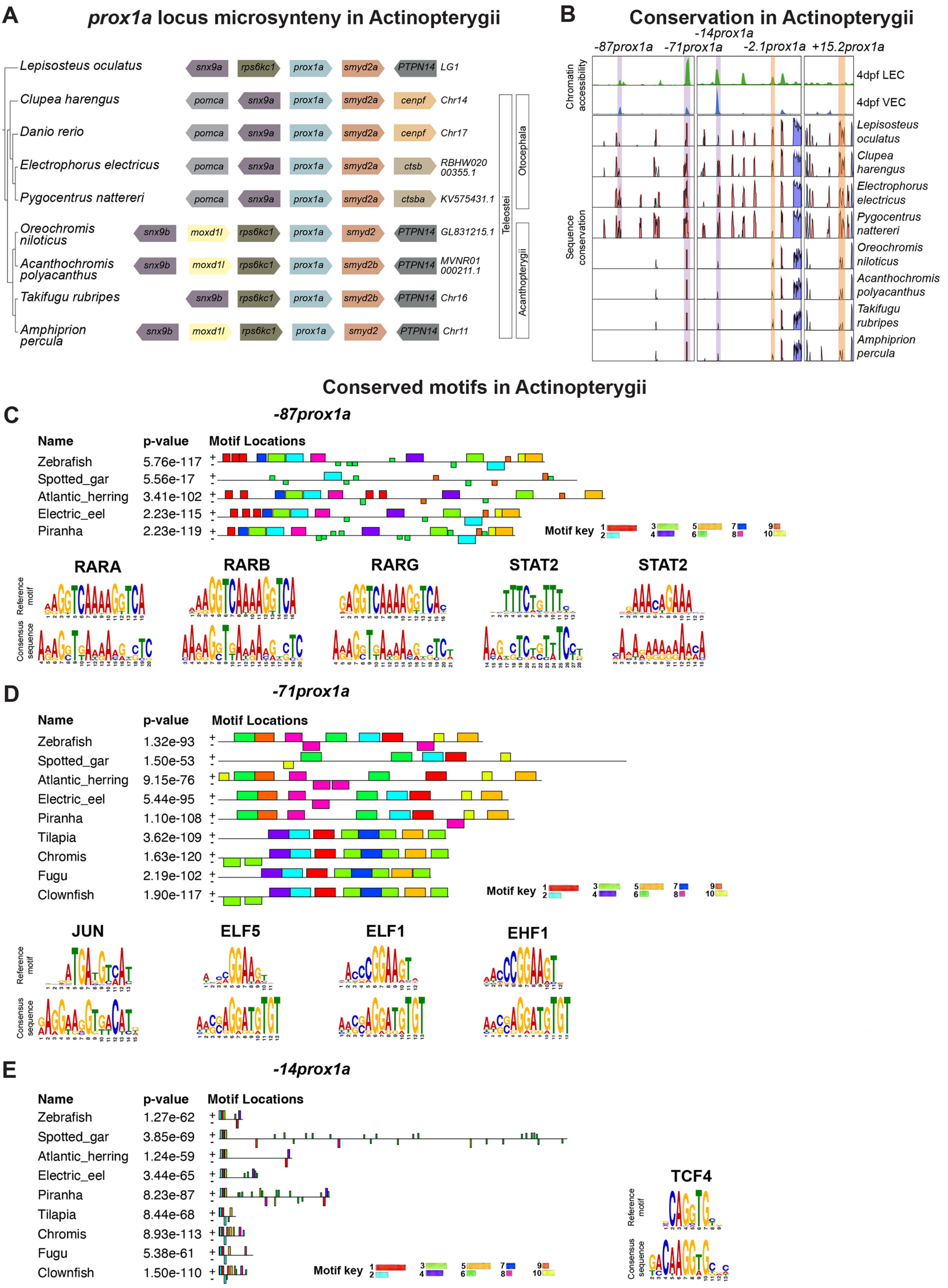

Development • Supplementary information

Fig. S4. *prox1a* microsynteny in Actinopterygii and predicted TFs binding to the snATAC-identified enhancers

(A) The microsynteny surrounding the Actinopterygii *prox1a* locus. Downstream of *prox1a*, *Smyd2a* is found in all analysed sequences. Upstream of *Prox1/prox1a, Rps6ck1* is found in *Lepisosteus oculatus* and Euteleostei, but not in Otocephala. The annotations used are listed in Table 1.

(B) Conservation analysis of the identified *prox1a* enhancers 9 Actinopterygii species. Results are shown compared to zebrafish. Blue peaks: exons; Red peaks: conserved non-coding DNA; Purple bars: snATAC-identified enhancers; Orange bar: conservation-identified enhances.

(C) Conserved motif structure and predicted conserved binding sites of the identified lymphatic *-87prox1a* enhancer in Actinopterygii. Motif locations are listed in Table S4 and S5.

(D) Conserved motif structure and predicted conserved binding sites of the identified lymphatic *-71prox1a* enhancer in Actinopterygii. Motif locations are listed in Table S4 and S5.

(E) Conserved motif structure and predicted conserved binding sites of the identified lymphatic *-14prox1a* enhancer in Actinopterygii. Motif locations are listed in Table S4 and S5.

Development • Supplementary information

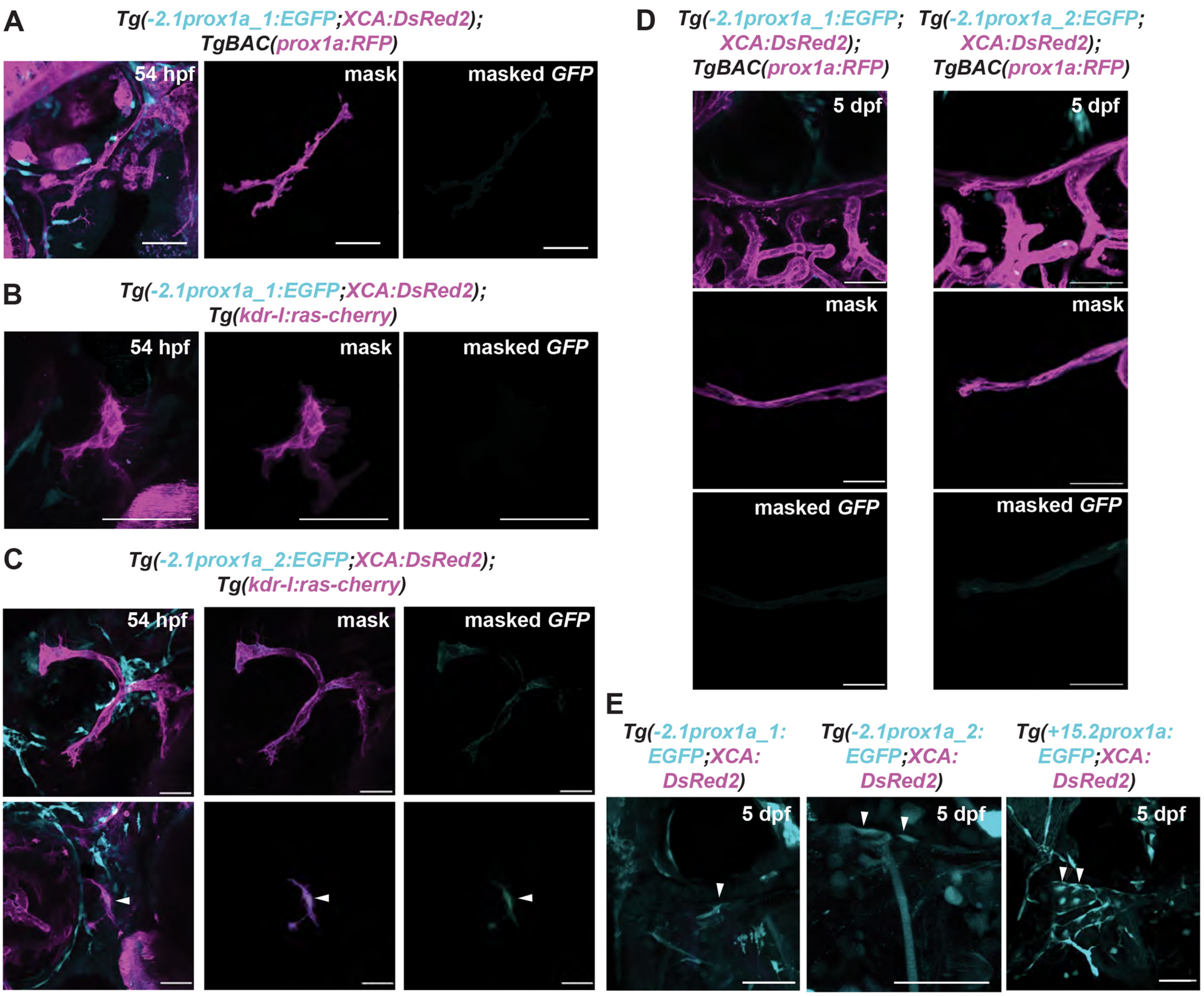

Fig. S5. Additional characterisation of the core element of *-2.1prox1a* activity

(A) Confocal projections of facial lymphatics labelled with *Tg(-2.1prox1a_1:EGFP; XCA:DsRed2)^{uu5kk}* (cyan) and *Tg(prox1a:RFP)^{nim5}* (magenta) at 54 hpf, showing no lymphatic expression.

(B) Confocal projections of VA-L labelled with *Tg(-2.1prox1a_1:EGFP;XCA:DsRed2)^{uu5kk}* (cyan) and *Tg(kdr-l:ras-cherry)^{s916}* (magenta) at 54 hpf, showing no lymphatic expression.

(C) Confocal projections of facial lymphatics labelled with *Tg(- 2.1prox1a_2:EGFP; XCA:DsRed2)^{uu6kk}* (cyan) and *Tg(kdr-l:ras-cherry)^{s916}* (magenta) at 54 hpf, showing -2.*1prox1a_2* driven expression in the VA-L (arrowhead).

(D) Confocal projections of the primary head sinus labelled with *Tg(- 2.1prox1a_1:EGFP; XCA:DsRed2)^{uu5kk}* and *Tg(-2.1prox1a_2:EGFP;XCA:DsRed2)^{uu6kk}* at 5 dpf, showing no expression.

(E) Enhancer-driven expression in *Tg(-2.1prox1a_1:EGFP;XCA:DsRed2)^{uu5kk}*, *Tg(- 2.1prox1a_2:EGFP;XCA:DsRed2)^{uu6kk}* and *Tg(+15.2prox1a:EGFP;XCA:DsRed2)^{uu7k}* in the facial lymphatics (arrowheads) at 5 dpf, excluding bleed-through as a source of the signal. Scales bars represent 50µm.

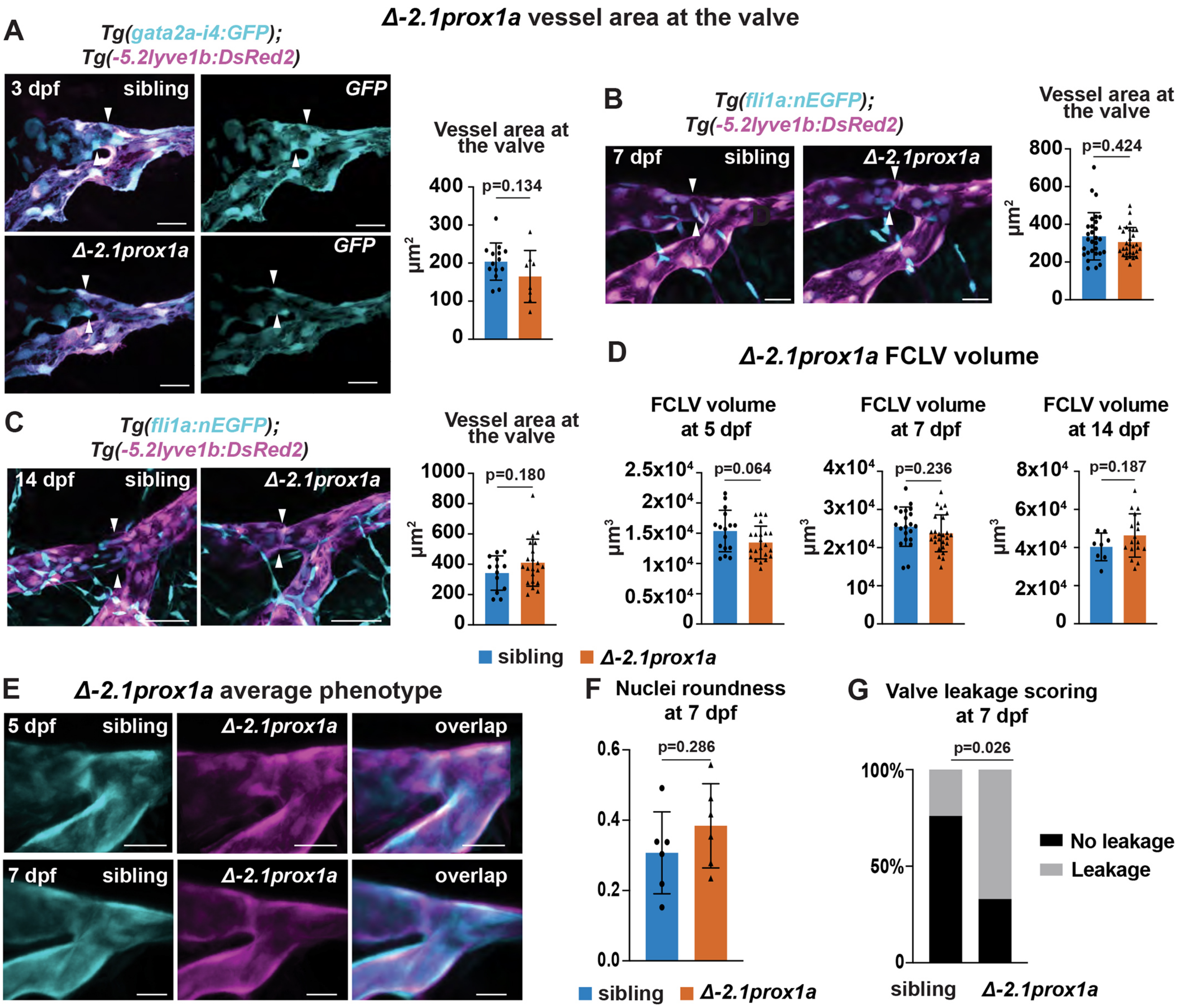
Δ-2.1prox1a vessel area at the valve
A
Tg(gata2a-i4:GFP);
Tg(-5.2lyve1b:DsRed2)
3 dpf
sibling
GFP
Δ-2.1prox1a
GFP
Vessel area at the valve
p=0.134
µm2
B
Tg(fli1a:nEGFP);
Tg(-5.2lyve1b:DsRed2)
7 dpf
sibling
Δ-2.1prox1a
Vessel area at the valve
p=0.424
C
Tg(fli1a:nEGFP);
Tg(-5.2lyve1b:DsRed2)
14 dpf
sibling
Δ-2.1prox1a
Vessel area at the valve
p=0.180
D
Δ-2.1prox1a FCLV volume
FCLV volume at 5 dpf
p=0.064
FCLV volume at 7 dpf
p=0.236
FCLV volume at 14 dpf
p=0.187
µm3
sibling
Δ-2.1prox1a
E
Δ-2.1prox1a average phenotype
5 dpf
7 dpf
overlap
F
Nuclei roundness at 7 dpf
p=0.286
G
Valve leakage scoring at 7 dpf
p=0.026
No leakage
Leakage
100%
50%

Fig. S6. Additional characterisation of Δ-*2.1prox1a* mutant embryos

(A) Confocal projections of *Tg(gata-i4:GFP)*^{uu11kk} *(cyan)* and *Tg(-5.2lyve1b:DsRed2)*^{nz101} (magenta) in sibling and Δ-*2.1prox1a* mutant embryos at 3 dpf. Quantification of vessel section at the valve (arrowheads) in Δ-*2.1prox1a* embryos at 3 dpf. Scale bar: 20µm. Mean ± sd, sibling (n=14) vs mutants (n=8): two-tailed Student's t-test, ns (p=0.134). 2 technical replicates, biological replicates correspond to the number of datapoints per condition.

(B) Confocal projections of *Tg(fli1:nEGFP)*^{y7} *(cyan)* and *Tg(-5.2lyve1b:DsRed2)*^{nz101} (magenta) in sibling and Δ-*2.1prox1a* mutant embryos at 7 dpf. Quantification of vessel section at the valve (arrowheads) in Δ-*2.1prox1a* embryos at 7 dpf. Scale bar: 50µm. Mean ± sd, sibling (n=29) vs mutants (n=29), two-tailed Mann-Whitney test, ns (p=0.424). 3 technical replicates, biological replicates correspond to the number of datapoints per condition.

(C) Confocal projections of *Tg(fli1:nEGFP)*^{y7} *(cyan)* and *Tg(-5.2lyve1b:DsRed2)*^{nz101} (magenta) in sibling and Δ-*2.1prox1a* mutant embryos at 14 dpf. Quantification of vessel section at the valve (arrowheads) in Δ-*2.1prox1a* embryos at 14 dpf. Scale bar: 50µm. Mean ± sd, sibling (n=13) vs mutants (n=21): two-tailed Student's t-test, ns (p=0.180). 3 technical replicates, biological replicates correspond to the number of datapoints per condition.

(D) Quantification of FCLV volume in Δ-*2.1prox1a* embryos at 5, 7 and 14 dpf. Mean ± sd. Left: two-tailed Student's t-test of 5 dpf siblings (n=16) vs Δ-*2.1prox1a* (n=22), ns (p=0.064). Centre: two-tailed Student's t-test of 7 dpf siblings (n=21) vs Δ-*2.1prox1a* (n=26), ns (p=0.236). Right: two-tailed Student's t-test of 14dpf siblings (n=8) vs Δ-*2.1prox1a* (n=17), ns (p=0.187). (E) Average valve phenotype in sibling (cyan) vs Δ-*2.1prox1a* (magenta) embryos at 5 dpf and 7 dpf, showing similar gross vessel morphology. Scale bar: 20µm.

(F) Quantification of sibling (n=6) vs Δ-*2.1prox1a* mutant (n=6) valve cell nuclei roundness in TEM-imaged embryos at 7 dpf. Mean ± sd. Representative images are present in Fig. 5D. two-tailed Student's t-test, ns (p=0.286). 2 technical replicates, biological replicates correspond to the number of datapoints per condition.

(G) Scoring of valve leakage in Qtracker injected 7 dpf siblings (n=21) vs Δ-*2.1prox1a* (n=12) embryos. Mean ± sd. Representative images are present in Fig. 5E. Fisher's test, p=0.026. 2 technical replicates, biological replicates correspond to the number of datapoints per condition.

Development • Supplementary information

Table S1. mVista analysis sequences

Available for download at
https://journals.biologists.com/dev/article-lookup/doi/10.1242/dev.202525#supplementary-data

Table S2. Tested *prox1a* putative CREs

Available for download at
https://journals.biologists.com/dev/article-lookup/doi/10.1242/dev.202525#supplementary-data

Table S3. Primers and gRNAs

Available for download at
https://journals.biologists.com/dev/article-lookup/doi/10.1242/dev.202525#supplementary-data

Table S4. Conserved motifs detected in evolutionary conserved enhancers

Available for download at
https://journals.biologists.com/dev/article-lookup/doi/10.1242/dev.202525#supplementary-data

Table S5. Predicted TFs binding sites in *prox1a* enhancers

Available for download at
https://journals.biologists.com/dev/article-lookup/doi/10.1242/dev.202525#supplementary-data

Table S6. Complete list motifs based on zebrafish (p-val < 1e-04)

Available for download at
https://journals.biologists.com/dev/article-lookup/doi/10.1242/dev.202525#supplementary-data

Table S7. GO terms enrichment in more and less accessible sequences in LECs

Available for download at
https://journals.biologists.com/dev/article-lookup/doi/10.1242/dev.202525#supplementary-data

Table S8. gRNAs used

Available for download at
https://journals.biologists.com/dev/article-lookup/doi/10.1242/dev.202525#supplementary-data

Additional files are uploaded containing tables separately

Table S5A. Predicted TFs binding sites in *prox1a* enhancers

Available for download at
https://journals.biologists.com/dev/article-lookup/doi/10.1242/dev.202525#supplementary-data

Table S6A. Complete list motifs based on zebrafish (p-val < 1e-04)

Available for download at
https://journals.biologists.com/dev/article-lookup/doi/10.1242/dev.202525#supplementary-data

Table S7A. GO terms enrichment in more and less accessible sequences in LECs

Available for download at
https://journals.biologists.com/dev/article-lookup/doi/10.1242/dev.202525#supplementary-data
