# Supplementary material for: Multiple cis-regulatory elements control prox1a expression in distinct lymphatic vascular beds
Source: Development. 2024 May 9;151(9):dev202525. doi: 10.1242/dev.202525 (PMC11128278; doi:10.1242/dev.202525)
Supplement: Supplementary file 1 [file develop-151-202525_review_history.pdf]

Multiple *cis*-regulatory elements control *prox1a* expression in distinct lymphatic vascular beds

Virginia Panara, Hujun Yu, Di Peng, Karin Staxäng, Monika Hodik, Beata Filipek-Gorniok, Jan Kazenwadel, Renae Skoczylas, Elizabeth Mason, Amin Allalou, Natasha L Harvey, Tatjana Haitina, Ben M Hogan and Katarzyna Koltowska
DOI: 10.1242/dev.202525

Editor: Benoit Bruneau

Review timeline
Submission to Review Commons: 24 August 2023
Submission to Development: 7 November 2023
Editorial decision: 9 January 2024
First revision received: 19 March 2024
Accepted: 21 March 2024

Reviewer 1

Evidence, reproducibility and clarity

Through the study of cis-regulatory element/enhancer activity in zebrafish, this study from Panara and colleagues provides insight into the transcriptional control of Prox1, a master regulator of lymphatic endothelial cell (LEC) fate. The authors used conservation of non-coding DNA, and chromatin accessibility data to identify enhancers that drive expression of a fluorescent reporter in anatomically distinct subsets of LECs. Analysis of transcription factor binding motifs in these enhancers suggests that differences in enhancer activity throughout the lymphatic vasculature may be due to binding of distinct transcription factors to these elements. Importantly, the authors identify a conserved 200 bp element within the -2.1kb enhancer that could drive expression in the lymphatic valve. Analysis of mutants carrying a 102 bp deletion in this region (including an Nfatc1 binding site), revealed reduced Prox1 expression and valve defects.

Major comments

- In the text it is suggested that sequence conservation was assessed across 8 species: "We aligned the region of the PROX1/prox1a locus in eight Osteichthyes species using mVISTA (Fig. 1A, Table S1)." Fig. 1A contains 7 species, and I am not able to find Table S1.
- It would be important to discuss reasons that the +15.2kb enhancer is not clearly identifiable in the scATAC-seq analyses but drives expression. Is this due to the relatively limited activity in facial lymphatics? Furthermore, given the degree of conservation, it would be useful to mutate specific transcription factor binding motifs (e.g. Mafb, Sox18, etc) in the -15.2kb enhancer and assess activity in the FCLV.
- OPTIONAL : Mutate Nr2f2 and Gata2 binding sites in -87kb enhancer to test for impact on activity. This would allow the authors to imply functional rather than sequence conservation. On a similar note, it would be interesting to understand if these enhancers are active in the context of mammalian lymphatic development.

© 2024. Published by The Company of Biologists under the terms of the Creative Commons Attribution License (https://creativecommons.org/licenses/by/4.0/).

Minor comments:
- It would be good to clarify in the following sentence that these enhancer marks are present at the whole embryo level and were not specifically identified in LECs : "Using zebrafish public databases for H3K4me1 and H3K27ac, we identified that ten of the selected prox1a CNEs were primed or active enhancers (Aday et al., 2011; Bogdanovic et al., 2012) (Fig. 1A, S1C)."
- "....(Gupta et al., 2007) to determine the motifs and putative transcription factor binding sides." - should read "....(Gupta et al., 2007) to determine the motifs and putative transcription factor binding sites".

- It might be more accurate to use zebrafish protein nomenclature for the transcription factor motifs in Fig1D, G and Fig2E (i.e. Gata2 not GATA2)

Significance

- While the roles of Prox1 in lymphatic vascular development and homeostasis are well established, relatively little is known about the cis-regulatory mechanisms governing it's expression; recent work has described an enhancer in the mouse Prox1 locus (Kazenwadel et al., 2023). This study from Panara and colleagues characterises numerous cis-regulatory elements in the zebrafish prox1a locus and demonstrates heterogeneous activity in anatomically distinct parts of the lymphatic vasculature. The imaging and characterisation of enhancer elements is of a high standard. Experiments that addressed the regulation of enhancers by upstream transcriptional or signalling cues would strengthen this study. Furthermore, to understand if there is functional conservation across evolution, analysis of activity in mouse embryos would be of interest.
- This should be of broad interest to the vascular biology and developmental biology fields.
- My expertise are in vascular biology, lymphatic development and developmental genetics.

Reviewer 2

Evidence, reproducibility and clarity

Panara et al. identified the enhancer regions necessary for the tissue- and organ-specific expression of Prox1a, which is essential for lymphatic vessel development in zebrafish. The authors compared the sequences of eight species of osteichthyes, identified Conserved Non-Coding Elements (CNEs), and further predicted active enhancers by combining public databases. This sequence was analyzed using the ZED system. As a result, they confirmed that two out of ten sequences caused GFP expression in a subset of lymphatic endothelial cells. +15.2prox1a was required for the prox1a expression in the facial collecting lymphatics, while -2.1prox1a was required in the facial lymphatic valves. They also predicted transcription factor binding to these enhancer regions and identified enhancer regions of -14, -71, -87Prox1a based on chromatin state. Additionally, they identified a core element within -2.1Prox1a, performed its loss-of-function experiments, and analyzed its phenotype, revealing the functional importance of these enhancer regions.
The paper is logically well-structured and informative concerning the expression control of Prox1a.

Major comments:

- Are the key conclusions convincing?

I think so.
- Should the authors qualify some of their claims as preliminary or speculative, or remove them altogether?

No need to qualify.
- Would additional experiments be essential to support the claims of the paper? Request additional experiments only where necessary for the paper as it is, and do not ask authors to open new lines of experimentation.

© 2024. Published by The Company of Biologists under the terms of the Creative Commons Attribution License (https://creativecommons.org/licenses/by/4.0/).

Figure 4H is probably incorrect. Looking at the morphology of the valve, it's inferred that the lymph flow goes from right to left. In this case, if the valve function is abnormal, the lymph flow that entered from the right should reflux back to the left. Is the reviewer misunderstanding something here? Clarity on this point could be provided with video images, similar to echocardiograms in mice.
- Are the suggested experiments realistic in terms of time and resources? It would help if you could add an estimated cost and time investment for substantial experiments.

Yes, they are realistic. I would like further clarification to ensure the data is correct.

Minor comments:

- Specific experimental issues that are easily addressable.

It's important where Prox1 is expressed in the lymphatic system, but if the identified enhancers also regulate Prox1 expression in other tissues like the myocardium, it would be a significant finding. Do these enhancers have a role in controlling Prox1 expression in lymphatics and non-lymphatics?

- Are prior studies referenced appropriately?

Yes.

- Are the text and figures clear and accurate?

Yes.
- Do you have suggestions that would help the authors improve the presentation of their data and conclusions?
1. Regarding Figure 4G, the actual image is unknown due to the red shading. Can you discuss or clarify how knocking out the Prox1 enhancer affects valve formation? If Prox1 expression is reduced, does the normal extracellular matrix change? Why do these morphological changes occur?
2. In mice, Prox1 is expressed in the heart valve endothelium. Have they checked the enhancer activity in other valves (heart valves or venous valves, if they exist)?
3. -2.1prox1a is said to be important for Prox1 expression in the facial lymphatic valves. Are valves only formed in this part of the lymphatics in zebrafish at this stage? Why is it important to identify the enhancer for this particular valve? Are there no other lymphatic valves? Please explain in the text.
4. They seem to emphasize +15.2's role in facial lymphatic expression. Compared to the trunk, why is it significant that there is a unique enhancer acting in this area? Is there something functionally or anatomically unique about facial lymphatics? Please discuss why the enhancer region's conservation is high in facial lymphatics.

Significance

- Describe the nature and significance of the advance (e.g. conceptual, technical, clinical) for the field.

In this paper, the authors have revealed previously unknown regulatory sequences of zebrafish Prox1a. They identified enhancer sequences that control Prox1a expression in the facial collecting lymphatics and lymphatic valves. The loss of the -2.1prox1a enhancer led to malformation in some lymphatic valves, suggesting the functional importance of this enhancer.
- State what audience might be interested in and influenced by the reported findings.

Readers interested in vascular development and those interested in transcriptional control during development.
- Define your field of expertise with a few keywords to help the authors contextualize your point of view. Indicate if there are any parts of the paper that you do not have sufficient

© 2024. Published by The Company of Biologists under the terms of the Creative Commons Attribution License (https://creativecommons.org/licenses/by/4.0/).

expertise to evaluate.

Lymphatic development in mammals, cardiac development, cardiology, cardiovascular pathology.

Reviewer 3

Evidence, reproducibility and clarity

This manuscript presents the cis-regulatory analysis of the enhancers controlling prox1a gene in zebrafish. Authors used both evolutionary conservation and existing single-cell ATAC data to highlight the major role of two elements. I feel that the transgenesis work is quite solid and the main conclusions interesting. However, I feel the authors need to provide some extra validations for some of the analysis.

1. the authors did not discuss the fact that euteleosts underwent an extra whole genome duplication and that prox1a might have a paralogue. They also perform genome alignment using non-duplicated outgroups (gar, xenopus) without discussing. I am a bit skeptical about the use of mVISTA on relatively short expert of sequence aroudn a gene, as it is not able to capture the global molecular evolution parameters. I think the authors should also examine some of the precomputed phastCons / phylocons data performed and available on UCSC to confirm their findings. probably they should also examien a few more fish genome. I don't find this evolutioanry analysis extremely convinced and careful - which doesn't mean that the conclusions are wrong.
2. I find the presentation, fairly obscure, the writing is quite convoluted, and the figures are very dense and not super explanatory, I would urge to improve (this is not helped by the fact that figure are their leged and presented at distinct places of this manuscript). For instance, I think having. a figure summarising signal from evolutionary conservation, scATAC and chromatin marks altogether would be quite essential.
3. I also find the reanalysis of the single-cell ATAC described too scarcely: which are the genes used to identify the different cell populations?
4. I feel the one additional experiment that the authors could have done would have been to use their construct to isolate the different cells population of interest and perform some regulatory profiling scuh as ATAC-seq or cut-and-tag on this population, to have a direct, in situ evidence of the activity of these regulatory elements.?

I also feel that the evolutionary aspect could be discussed a bit more, what are the differences between the diffeerent vertebrate lineage, etc...

(p7) active enhancer in a tissue: while ATAC gives a good indicated of accessibility it is not an indicate of activity as for instance H3K27Ac would be.

Significance

I think this is an interesting piece of work, which elaborates on previous studies on prox1a involvment in the lymphatic system but it doesn not bring essentially new perspective on the question.

Author response to reviewers' comments

Manuscript number: RC-2023-02142
Corresponding author(s): Katarzyna, Koltowska

1. General Statements

This study is of significant scientific importance as it determines the intricate regulatory aspects of PROX1 in lymphatic vasculature development. The onset of Prox1 expression is considered the

© 2024. Published by The Company of Biologists under the terms of the Creative Commons Attribution License (https://creativecommons.org/licenses/by/4.0/).

first indicator of specified lymphatic endothelial cells that differentiates this cell population from the blood endothelial cell identity and is central to lymphatic development. Despite decades of research, the transcriptional cohort underlying the induction of Prox1 expression remains unknown.
Using a conservation approach in conjunction with chromatin accessibility determined by single nuclei ATAC-sequencing to identify regulatory elements driving *prox1* expression in the lymphatic endothelium. A key discovery of this study is the identification of five distinct regulatory elements capable of driving *prox1a* expression in different subsets of lymphatic vasculature. Mechanistically, we uncovered that non-conserved distal regulatory elements induce *prox1* expression in a pan-lymphatic manner. Conversely, the proximal elements were conserved and induced restricted expression patterns in specific subsets of the lymphatic endothelium. This suggests that *prox1a* expression in lymphatics is under nuanced regulation, contingent on anatomical position and vessel function.
To test the functional relevance, we characterised the phenotype resulting from the deletion of a lymphatic valve-specific enhancer, and found that both valve morphology and functionality are compromised, shedding light on the significance of this differential regulation in shaping the development of specific lymphatic vessel segments.
In summary, this research represents a significant step towards a comprehensive understanding of how a key transcription factor, PROX1, is regulated in the context of lymphatic vasculature development. Its scientific importance is not limited to vascular biology but extends to broader areas of medical and biological research. Given the mechanistic insights into our understanding of Prox1 regulation and the lymphatic vasculature development, this work undoubtedly deserves publication in a journal with a broad readership.

We would like to thank the reviewers for their insightful comments which has helped us improve our manuscript and identify further experiments needed to strengthen our conclusions.
Please find the reviewers comments below in blue and our responses in black. Modifications in the manuscript are highlighted in grey.

2. Description of the planned revisions

Reviewer 1: It would be important to discuss reasons that the +15.2kb enhancer is not clearly identifiable in the scATAC-seq analyses but drives expression. Is this due to the relatively limited activity in facial lymphatics? Furthermore, given the degree of conservation, it would be useful to mutate specific transcription factor binding motifs (e.g. Mafb, Sox18, etc) in the -15.2kb enhancer and assess activity in the FCLV.

We thank Reviewer 1 for this comment. ATAC-seq was performed at 4 dpf, which provides only a snapshot of chromatin activity. The lack of opening signature at *+15.2prox1a* could be due to the dynamic activity of this enhancer. We have checked publicly available data set for H3K27ac and H3K4me1 and in long-pec and adults this enhancer is marked as either primed or active. Therefore, we speculate that the enhancer might have a strong temporal dependence. Since we did not use destabilised GFP, the reporter expression does not reflect this. We have now added a sentence in the paper addressing this possibility: *“The +15.2prox1a enhancer does not appear to be accessible in either LECs or BECs at 4 dpf, which could be explained by dynamic activity of this element.”*
To evaluate the functional relevance of the predicted binding sites for known lymphatic regulators (Sox18, Mafb, and Gata2) to this enhancer, we will generate a mutant version of this vector in which such sites are disrupted. The mutant and WT vectors will be injected and the intensity of the expression evaluated in mosaic F0 to establish if the TF binding sites are necessary for enhancer activity.

Reviewer 1: OPTIONAL: Mutate Nr2f2 and Gata2 binding sites in -87kb enhancer to test for impact on activity. This would allow the authors to imply functional rather than sequence conservation. On a similar note, it would be interesting to understand if these enhancers are active in the context of mammalian lymphatic development.

Addressing the activity of the enhancers in mammals, it is important to note that out of the five described enhancers, only two are conserved in mammals and can therefore easily be compared: *-2.1prox1a* and *+15.2prox1a*. *-2.1prox1a* activity in mouse lymphatics has been extensively

© 2024. Published by The Company of Biologists under the terms of the Creative Commons Attribution License (https://creativecommons.org/licenses/by/4.0/).

characterised by Kazenwadel et al. (2023). Unfortunately, no datasets (either ATAC, histone modifications, or DNAse sensitivity) are currently available for mammalian lymphatic cells during developmental stages. This is particularly relevant for *+15.2prox1a*, an enhancer which is active very early in lymphatic development in zebrafish. Therefore, it is not possible to assess the activity of *+15.2prox1a* in mammalian lymphatic cells using in silico tools.
We will address the upstream regulation of *-87prox1a* by known lymphatic regulators in mammals (such as Nr2f2 and Gata2) by injecting a mutant version of the enhancer in which the binding sites are disrupted and quantify the effect on enhancer activity, as described in the previous comment.

Reviewer 2: It's important where Prox1 is expressed in the lymphatic system, but if the identified enhancers also regulate Prox1 expression in other tissues like the myocardium, it would be a significant finding. Do these enhancers have a role in controlling Prox1 expression in lymphatics and non-lymphatics? (Planned revision)

We thank Reviewer 2 for this suggestion. We have added whole-body images of GFP expression driven by *-2.1prox1a* in a 5 days post fertilization embryo. Notably, this lymphatic valve enhancer was not active in the heart or cardiac valves (Figure S1L). Similar images for the remaining enhancers will be generated during revision to fully assess the enhancers activity in other tissues, including the myocardium.

3. Description of the revisions that have already been incorporated in the transferred manuscript

Reviewer 1: In the text it is suggested that sequence conservation was assessed across 8 species: "We aligned the region of the PROX1/prox1a locus in eight Osteichthyes species using mVISTA (Fig. 1A, Table S1)." Fig. 1A contains 7 species, and I am not able to find Table S1.

We apologise for the confusion caused by our wording. The conservation analysis includes seven vertebrate species which are aligned to zebrafish, making it 8 species in total. We have modified the text to clarify how the analysis was performed. "*To identify enhancer elements in the zebrafish prox1a locus, we analysed DNA conservation, as Conserved Non-coding Elements (CNE) close to a gene can indicate the presence of enhancers. We aligned the region of the PROX1/prox1a locus of seven Osteichthyes species against the zebrafish one using mVISTA.*" All supplementary tables will be provided upon acceptance of the manuscript for publication.

Reviewer 1: It would be good to clarify in the following sentence that these enhancer marks are present at the whole embryo level and were not specifically identified in LECs : "Using zebrafish public databases for H3K4me1 and H3K27ac, we identified that ten of the selected prox1a CNEs were primed or active enhancers (Aday et al., 2011; Bogdanovic et al., 2012) (Fig. 1A, S1C)."

We thank Reviewer 1 for pointing out this oversight. The text has been modified to better highlight the use of whole-embryo datasets: "*Currently, no LEC-specific zebrafish databases for H3K4me1 and H3K27ac are available. Whole-body databases were instead used to identified that ten of the selected prox1a CNEs were primed or active enhancers*"

Reviewer 1: - "....(Gupta et al., 2007) to determine the motifs and putative transcription factor binding sides." - should read "....(Gupta et al., 2007) to determine the motifs and putative transcription factor binding sites"

We thank Reviewer 1 for catching the typo, and the text has been modified accordingly.

Reviewer 2: Figure 4H is probably incorrect. Looking at the morphology of the valve, it's inferred that the lymph flow goes from right to left. In this case, if the valve function is abnormal, the lymph flow that entered from the right should reflux back to the left. Is the reviewer misunderstanding something here? Clarity on this point could be provided with video images, similar to echocardiograms in mice.

For the valve experiment, we referred to Shin et al. (2019). Briefly, the zebrafish embryos are anaesthetised with tricaine before the injection takes place. Tricaine anaesthesia causes the valve to close shut, not open. Therefore, in normal condition none of the injected tracker should be observed past the valve position. We have modified the text to explain this more clearly as we

© 2024. Published by The Company of Biologists under the terms of the Creative Commons Attribution License (https://creativecommons.org/licenses/by/4.0/).

realise it could create confusion for readers: “*To assess whether the altered morphology could affect valve function, we performed Qtracker injections in the LFL in anaesthetised 7 dpf larvae as previously described (Shin et al., 2019). Briefly, a correctly formed valve should be closed under tricaine anaesthesia, preventing the Qtracker from moving into the FCLV.*”

Reviewer 2: It's important where Prox1 is expressed in the lymphatic system, but if the identified enhancers also regulate Prox1 expression in other tissues like the myocardium, it would be a significant finding. Do these enhancers have a role in controlling Prox1 expression in lymphatics and non-lymphatics? (Incorporated experiment)

We have added whole-body images of GFP expression driven by *-2.1prox1a* in a 5 days post-fertilisation embryo, in order to show the overall pattern of expression. As described above, similar images for the remaining enhancers will be generated during revision.

Reviewer 2: Regarding Figure 4G, the actual image is unknown due to the red shading. Can you discuss or clarify how knocking out the Prox1 enhancer affects valve formation? If Prox1 expression is reduced, does the normal extracellular matrix change? Why do these morphological changes occur?

We thank Reviewer 2 for pointing this out. We have modified Figure 4 by adding the unshaded version of the EM images. We have also integrated a point summarising the known regulation of the leaflet extracellular matrix by PROX1 in the discussion: “*The -2.1prox1a mutants are viable and fertile and only show developmental defects in the lymphatic valve regarding the morphology of the leaflets. Key factors in the regulation of the leaflet extracellular matrix such as integrin-a9 (Bazigou et al., 2009) have been suggested to be regulated under the Prox1/Foxc/Nfatc1/Gata2 network in zebrafish lymphatic valve formation (Shin et al., 2019), which could explain the observed phenotype.*”

Reviewer 2: In mice, Prox1 is expressed in the heart valve endothelium. Have they checked the enhancer activity in other valves (heart valves or venous valves, if they exist)?
Reviewer 2: -2.1prox1a is said to be important for Prox1 expression in the facial lymphatic valves. Are valves only formed in this part of the lymphatics in zebrafish at this stage? Why is it important to identify the enhancer for this particular valve? Are there no other lymphatic valves? Please explain in the text.

In the late embryonic and early larval stages of zebrafish, the facial lymphatic valve is the only lymphatic valve which has been described so far. Following Reviewer 2’s suggestions, we additionally characterised the activity of the *-2.1prox1a* enhancer in the other vascular valves present in zebrafish at 5 dpf. These include two lymph-venous valves in the face as well as in the developing atrioventricular and bulboventricular cardiac valves. The results have been integrated into Supplementary Figure 1 and the text of the manuscript: “*We additionally investigated enhancer activity in other vascular valves present during embryonic development. Two lymphovenous valves (LVVs) have been described to connect the facial lymphatics to the veins (Meng et al., 2023; Shin et al., 2019). We detected weak activity of -2.1prox1a in the anterior LVV connecting the FCLV to the PHS, but none in the posterior LVV. No activity was detected in association with the developing atrio-ventricular and bulbo-ventricular cardiac valves. Whole-body imaging at 5 dpf revealed additional expression only in a population of cells in the abdomen and extremely weak expression in few muscle cells.*”

Reviewer 2: They seem to emphasize +15.2's role in facial lymphatic expression. Compared to the trunk, why is it significant that there is a unique enhancer acting in this area? Is there something functionally or anatomically unique about facial lymphatics? Please discuss why the enhancer region's conservation is high in facial lymphatics.

We thank Reviewer 2 for pointing out the missing information. We have added references in the discussion explaining the unique regulation involved in the formation of the FCLV and lymphatic valve compared with the rest of the lymphatic vasculature. “*Two of the enhancers, -2.1prox1a and +15.2prox1a, show significantly enriched expression respectively in the valve and the FCLV. These anatomically and functionally distinct subsets of the lymphatics are also regulated by different networks from the remaining lymphatics (Hußmann et al., 2023; Meng et al., 2023; Shin et al.,*

© 2024. Published by The Company of Biologists under the terms of the Creative Commons Attribution License (https://creativecommons.org/licenses/by/4.0/).

2019).”

Reviewer 3: Probably they should also examien a few more fish genome. I don't find this evolutioanry analysis extremely convinced and careful - which doesn't mean that the conclusions are wrong

The conservation analysis is shown in Figure 1 (and Suppl. Fig. 1) was used to identify candidate enhancers conserved between mammals and zebrafish, and therefore included only a few Actinopterygii species. However, a second conservation analysis was performed (Figure 2) with the objective of better understanding the conservation and/or loss of the identified prox1a enhancers within the actinopterygian clade. This analysis includes nine Actinopterygii species selected with a good spread within the group; however, if the reviewer would like to suggest additional fish genomes that should be included to strengthen our analysis, we would be happy to modify it.
In addition, we have added the *+15.2prox1a* enhancer to the panel, and the conservation of *-2.1prox1a* and *+15.2prox1a* within Actinopterygii has been addressed in the text: “*As expected, both -2.1prox1a and +15.2prox1a are conserved across Actinopterygii*.”

Reviewer 3: They also perform genome alignment using non-duplicated outgroups (gar, xenopus) without discussing. I am a bit skeptical about the use of mVISTA on relatively short expert of sequence aroudn a gene, as it is not able to capture the global molecular evolution parameters. I think the authors should also examine some of the precomputed phastCons / phylocons data performed and available on UCSC to confirm their findings.

We thank Reviewer 3 for this comment. We have added an additional panel in Suppl. Figure 1 and Suppl. Figure 2 showing the conservation of the identified enhancers across vertebrates and actinopterygians using PhyloP and the Multiz vertebrate alignment tracks on UCSC. The text has been modified accordingly:
“The conservation of the identified elements was further confirmed using PhyloP and the Multiz alignment to vertebrate tracks in the UCSC Genome browser”
“The reduced level of conservation of -87prox1a and -14prox1a also explains why only -71prox1a is marked as conserved in the UCSC Genome Browser Multiz Alignment”

Reviewer 3: I find the presentation, fairly obscure, the writing is quite convoluted, and the figures are very dense and not super explanatory, I would urge to improve (this is not helped by the fact that figure are their leged and presented at distinct places of this manuscript). For instance, I think having. a figure summarising signal from evolutionary conservation, scATAC and chromatin marks altogether would be quite essential.

We thank Reviewer 3 for these observations. We have modified our figures by adding titles to the different subsections, which we hope has increased the readability of the described results. We have also inserted a summarising panel showing in one place all the relevant data for the five enhancers we identified in Figure 3. The panel includes the snATAC signal, evolutionary conservation analysis, and available chromatin markers. However, no good lymphatic (nor endothelial) histone modification database is currently available, and therefore the represented data refer to whole-body data from earlier stages. For this reason, H3K4me1 (marking primed enhancers) was considered together with H3K27ac, which marks active enhancers.

Reviewer 3: I also find the reanalysis of the single-cell ATAC described too scarcely: which are the genes used to identify the different cell populations

We have added more details in the methods section about how the different populations have been identified in the scATAC analysis: “*Briefly, the LEC cluster was defined by combined high accessibility at the prox1a, cdh6, and lyve1b loci. The VEC clusters by combined high accessibility at the cdh5, kdrl, and stab2 or lyve1b loci, and AECs by combined high accessibility at the cdh5, kdrl, flt1, and dll4 loci but low accessibility at lybe1b. MuLECs were identified based on the high accessibility at osr2 in addition to the standard LEC loci but low accessibility at cdh6, and the endocardium by the accessibility at the cdh5 and hand2 loci.*”

Reviewer 3: I also feel that the evolutionary aspect could be discussed a bit more, what are the differences between the diffeerent vertebrate lineage, etc...

© 2024. Published by The Company of Biologists under the terms of the Creative Commons Attribution License (https://creativecommons.org/licenses/by/4.0/).

We have added a passage in the text that addressed the known molecular similarities in the lymphatic development of zebrafish and mammals, such as growth factor signalling and transcription factors, as well as the obvious molecular differences, such as the presence of only one valve in zebrafish, which highlights how interesting it is that we found such strongly conserved lymphatic valve enhancers. "*Despite obvious morphological differences, the molecular program underlying LEC development in mammals and zebrafish presents strong similarities, such as the role of growth factors signalling and TFs such as PROX1, NFATC1 or GATA2 (Kazenwadel et al., 2015; Koltowska et al., 2015a; Norrmén et al., 2009; Secker and Harvey, 2021; Shin et al., 2019; Wigle and Oliver, 1999). Therefore, it is worth noticing that the majority of the binding sites for these conserved lymphatic regulators are found in the sequence conserved enhancers.*"

Reviewer 3: (p7) active enhancer in a tissue: while ATAC gives a good indicated of accessibility it is not an indicate of activity as for instance H3K27Ac would be.

We agree with Reviewer 3 that specific histone modifications (such as H3K4me1 or H3K27ac) are more reliable indicators of enhancer identity than simple chromatin accessibility. However, no dataset mapping such histone modifications in lymphatic or endothelial cells in zebrafish exists owing to technical limitations. Therefore, in this study, we used the best available method (scATAC), which allows the identification of both enhancers and silencers. In the Results section, we clearly explain why we were limited in using histone modifications as a proxy for enhancer identity: "Histone modifications have been linked to non-coding elements such as promoters and enhancers. Specifically, H3K4me1 marks primed and active enhancers (Heintzman et al., 2007), whereas H3K27ac marks active enhancers (Bonn et al., 2012; Creyghton et al., 2010). Currently, no LEC-specific zebrafish databases are available for H3K4me1 and H3K27ac. Whole-body databases were instead used to identified ten of the selected *prox1a* CNEs as primed enhancers"

4. Description of analyses that authors prefer not to carry out

Reviewer 1: It might be more accurate to use zebrafish protein nomenclature for the transcription factor motifs in Fig1D, G and Fig2E (i.e. Gata2 not GATA2)

It is true that the currently used nomenclature is human. However, this reflects the databases used for transcription factor prediction. Currently, the best available databases for vertebrate transcription factor predictions are based on mice and humans, and the nomenclature reflects this fact.

Reviewer 3: The authors did not discuss the fact that euteleosts underwent an extra whole genome duplication and that prox1a might have a paralogue.

We apologise for the lack of clarity. In Suppl. Figure 1 shows the analysis of the gene that has been previously identified in the literature as the *prox1a* paralogue, called *prox3* or *prox1b*. Briefly, this figure shows the sequence conservation analysis, which did not reveal any promising candidates.

Reviewer 3: I feel the one additional experiment that the authors could have done would have been to use their construct to isolate the different cells population of interest and perform some regulatory profiling scuh as ATAC-seq or cut-and-tag on this population, to have a direct, in situ evidence of the activity of these regulatory elements.?

Such an experiment would doubtlessly provide important insight into the specific molecular signature associated, for example, with the valve-forming cells compared with regular lymphatic endothelial cells, and the lines we generated in this study would be great tools to answer these questions. However, the goal of this study was to identify *prox1a* enhancers responsible for driving the expression of this key factor in the lymphatic endothelium. Nonetheless, we would be glad to provide the vectors we generated to any laboratory interested in running such an experiment, and we do not exclude that we might look into these aspects ourselves in the future.

© 2024. Published by The Company of Biologists under the terms of the Creative Commons Attribution License (https://creativecommons.org/licenses/by/4.0/).

Original submission

First decision letter

MS ID#: DEVELOP/2023/202525

MS TITLE: Multiple cis-regulatory elements control prox1a expression in distinct lymphatic vascular beds

AUTHORS: Virginia Panara, Hujun Yu, Di Peng, Karin Staxang, Monika Hodik, Beata Filipek-Gorniok, Jan Kazenwadel, Renae Skoczylas, Elizabeth A Mason, Amin Allalou, Natasha Harvey, Tatjana Haitina, Ben L Hogan, and Katarzyna Koltowska

I have now received all the referees reports on the above manuscript, and have reached a decision. The referees' comments are appended below, or you can access them online: please go to BenchPress and click on the 'Manuscripts with Decisions' queue in the Author Area.

Based on the revision plan I plan to accept your manuscript. I did feel the need to secure an additional opinion, which you will see brings up issues of relevance, which would be important to address in a revised manuscript. Please attend to all of the reviewers' comments in your revised manuscript and detail them in your point-by-point response. If you do not agree with any of their criticisms or suggestions explain clearly why this is so. If it would be helpful, you are welcome to contact us to discuss your revision in greater detail. Please send us a point-by-point response indicating your plans for addressing the referees’ comments, and we will look over this and provide further guidance.

Reviewer 1

Advance summary and potential significance to field

In this study the authors have identified and characterised a series of conserved enhancers acting on the prox1a locus in zebrafish, to identify cis-regulatory sequences that drive expression in specific lymphatic beds within the developing zebrafish embryo. They aligned sequences across Osteichthyes spp against zebrafish, interrogated existing H3K4me1 and H3K27ac databases and then tested the ability of the identified enhancer sequences to drive GFP-reporter expression in larval stages. They observed two elements located downstream and upstream of the prox1a TSS: +15.2 prox1a and -2.1prox1a, which drove expression in the facial collecting lymphatic vessels and the lymphatic valve, respectively. Each enhancer sequence contained predicted binding sites for well characterised TFs previously implicated in vessel development including NFATC1, FOX, GATA2, MAFB, SOX18, PROX1 and KLF4. Analysis of a previously generated single cell ATAC-Seq dataset of zebrafish ECs, confirmed an open region of chromatin that mapped to the -2.1prox1a enhancer with an additional 219bp sequence and identified six regions in total, of which three: 87prox1a, -71prox1a, -14prox1a, with varying degrees of cross-species conservation, were confirmed to drive reporter gene expression in the lymphatic endothelium in stable lines. The authors then tested the additional 3’ sequence of -2.1prox1a arising from the scATAC-seq and identified facial lymphatic activity. Finally, they used CRISPR/Cas9 editing to delete 102 bp of -2.1prox1a from the prox1a locus, containing the predicted NFATC1 binding site, and observed normal facial lymphatic vessel morphology, but reduced length of the valve leaflets and altered valve morphology between 7 and 14dpf, to conclude the importance of this enhancer for valve development and prox1a function in the valve.

Currently there is very little known about the upstream regulation of Prox1/PROX1 as a master regulator of lymphatic endothelial cell fate. Sox18 and COUPTF have been implicated in the mouse, alongside the identification of a single Prox1 enhancer, but this does not explain the full regulation of Prox1/PROX1 expression and function in the various lymphatic beds during development. This study sheds some light on this by identifying further cis-regulatory enhancer regions in the zebrafish orthologue prox1a, which reveal conservation across ray-finned fish and some micro-conservation of sequences (microsynteny) with tetrapods.

© 2024. Published by The Company of Biologists under the terms of the Creative Commons Attribution License (https://creativecommons.org/licenses/by/4.0/).

Comments for the author

The studies as described are relatively preliminary, in terms of utilising existing databases to identify conserved cis-acting sequences, revealing lymphatic reporter gene expression in distinct vessel beds and a potential function for a 102 bp sequence in lymphatic valve development. The study does not really address its stated central aim, to identify the upstream regulation of prox1a in lymphatics. Each of the identified cis-regulatory elements, and most notably the -2.1prox1a element, is assigned a series of putative TF binding sites, predicted by in silico analyses, many of which have been implicated in vessel development. These TFs are not tested in the context of directly regulating prox1a expression, via a more site-specific mutagenesis and/or direct targeting of TFs using a LEC-conditional deletion approach. As such the study falls short of providing insights into the molecular regulation of prox1a and identifying specific pathways that may regulate its function(s) across lymphatic vascular beds. In addition, spatial resolution is lacking with regards to whether the elements identified, and putative regulatory TFs influence LEC fate versus lymphatic vessel formation, or morphogenesis. Finally, extrapolating from ray-finned fish the relevance to mammalian Prox1 regulation is unclear; the authors describe some microsynteny, but do not test these regions in the context of mammalian (eg. mouse) conservation.

More specific comments:
1. Figures 1-4 and relevant Supp Figures: inclusion of low-resolution images would be helpful to orientate the high-resolution panels (C. E and F); in terms of anatomical localisation of the reporter expression relative to the whole embryo at the developmental stages shown.

2. Figure 4G: the EM images show dysmorphic valves in delta-2.1prox1a mutants, but it is unclear on the extent of variation here and penetrance of phenotype(?). In addition, do the authors have a view as to whether the phenotype is underpinned by shorter cell length or is this a morphogenesis phenotype? More importantly, what do the authors think is the role of prox1a specifically in this context of valve development?

Some speculation in the Discussion here would be a useful addition.
3. Figure 4H. The qTracker images are difficult to reconcile as having observable differences, and the explanation of these panels (with respect to valve function in sibling control v mutant fish) is lacking in the text (page 11).
4. What is the functional significance of conserved regions of microsynteny of the zebrafish prox1a enhancers (most notably +15.2 prox1a and -2.1prox1a) with the regulation of mammalian/murine Prox1?
5. In the targeted 102 bp spanning the putative NFATC1 binding site (in the -2.1prox1a enhancer) what is the predicted additional TF binding? How does targeting the NFATC1 site alone, versus neighbouring sequences, affect prox1a expression in the valve versus valve morphogenesis? This gets at the more general comment above and the need to define upstream TF regulation of prox1a expression/function.

First revision

<u>Author response to reviewers' comments</u>

Response to reviewers

We would like to thank the reviewers for their insightful comments which has helped us improve our manuscript and identify further experiments needed to strengthen our conclusions.
Please find the reviewers comments below in blue and our responses in black. In the manuscript file, modifications to the text are also highlighted in grey to facilitate the reviewing process.

© 2024. Published by The Company of Biologists under the terms of the Creative Commons Attribution License (https://creativecommons.org/licenses/by/4.0/).

Reviewer 1 (Review commons)

Reviewer 1: It would be important to discuss reasons that the +15.2kb enhancer is not clearly identifiable in the scATAC-seq analyses but drives expression. Is this due to the relatively limited activity in facial lymphatics? Furthermore, given the degree of conservation, it would be useful to mutate specific transcription factor binding motifs (e.g. Mafb, Sox18, etc) in the -15.2kb enhancer and assess activity in the FCLV.

ATAC-seq was performed at 4 dpf, which provides only a snapshot of chromatin activity. The lack of opening signature at *+15.2prox1a* could be due to the dynamic activity of this enhancer. We have checked publicly available data set for H3K27ac and H3K4me1 and in long-pec and adults this enhancer is marked as either primed or active. Therefore, we speculate that the enhancer might have a strong temporal dependence. Since we did not use destabilised GFP, the reporter expression does not reflect this. We have now added a sentence in the paper addressing this possibility: *"The +15.2prox1a enhancer does not appear to be accessible in either LECs or BECs at 4 dpf, which could be due to a dynamic activity of this element."*

To evaluate the functional relevance of the predicted binding sites for known lymphatic regulators (Sox18, Mafb, and Gata2) to this enhancer we have assessed its expression level in the *mafba* single and *mafba/mafbb* double mutants. This experimental set up allows us to determine if the expression induced by *+15.2prox1a* was affected by the loss of Mafb transcription factors. We found a reduction of activity in *mafba* mutants, which is even more severe in double mutants. The data has been addressed in the main text *"In order to test the functional importance of the predicted binding sites, we took advantage of the mafba^{uq4bh} and mafbb^{ub47bh} mutant lines available in our lab (Arnold et al., 2022; Koltowska et al., 2015b). Quantification of the activity of +15.2prox1a in the mutant lines revealed a 60% reduction in enhancer activity in mafba mutants and 85% in double mutants (Fig. 1E). This confirms the functional relevance of the MAFB predicted binding site for +15.2prox1a activity, and suggests this enhancer is an important driver of prox1a contributing to the regulatory logic necessary for its correct spatial expression in developing lymphatics."*

Reviewer 1: OPTIONAL: Mutate Nr2f2 and Gata2 binding sites in -87kb enhancer to test for impact on activity. This would allow the authors to imply functional rather than sequence conservation. On a similar note, it would be interesting to understand if these enhancers are active in the context of mammalian lymphatic development.

It is important to note that out of the five described enhancers, only two are conserved in mammals and can therefore easily be compared: *-2.1prox1a* and *+15.2prox1a*. *- 2.1prox1a* activity in mouse lymphatics has been extensively characterised by Kazenwadel et al. (2023). Unfortunately, no datasets (either ATAC, histone modifications, or DNAse sensitivity) are currently available for mammalian lymphatic cells during developmental stages. This is particularly relevant for *+15.2prox1a*, an enhancer which is active very early in lymphatic development in zebrafish. Therefore, it is not possible to assess the activity of *+15.2prox1a* in mammalian lymphatic cells using *in silico* tools.

We attempted to address the relevance of the proposed TFs by injecting a mutant version of the -87kb enhancer in which the binding sites are disrupted and quantify the effect on enhancer activity. However, such injections failed to return embryos consistently expressing the reporter expression in the WT injected embryos. We think this might be due to weak transient expression of these constructs. Due to revision time constrained we could not generate stable lines.

Reviewer 1: In the text it is suggested that sequence conservation was assessed across 8 species: "We aligned the region of the PROX1/prox1a locus in eight Osteichthyes species using mVISTA (Fig. 1A, Table S1)." Fig. 1A contains 7 species, and I am not able to find Table S1.

We apologise for the confusion caused by our wording. The conservation analysis includes seven vertebrate species which are aligned to zebrafish, making it 8 species in total. We have modified the text to clarify how the analysis was performed. *"To identify enhancer elements in the zebrafish prox1a locus, we analysed DNA conservation, as Conserved Non-coding Elements (CNE) close to a gene can indicate the presence of enhancers. We aligned the region of the PROX1/prox1a locus of seven Osteichthyes species against the zebrafish one using mVISTA."* All

© 2024. Published by The Company of Biologists under the terms of the Creative Commons Attribution License (https://creativecommons.org/licenses/by/4.0/).

supplementary tables will be provided upon acceptance of the manuscript for publication.

Reviewer 1: It would be good to clarify in the following sentence that these enhancer marks are present at the whole embryo level and were not specifically identified in LECs : "Using zebrafish public databases for H3K4me1 and H3K27ac, we identified that ten of the selected prox1a CNEs were primed or active enhancers (Aday et al., 2011; Bogdanovic et al., 2012) (Fig. 1A, S1C)."

We thank Reviewer 1 for pointing out this oversight. The text has been modified to better highlight the use of whole-embryo datasets: "*Currently, no LEC-specific zebrafish databases for H3K4me1 and H3K27ac are available. Whole-body databases were instead used to identified that ten of the selected prox1a CNEs were primed or active enhancers*".

Reviewer 1: - "....(Gupta et al., 2007) to determine the motifs and putative transcription factor binding sides." - should read "....(Gupta et al., 2007) to determine the motifs and putative transcription factor binding sites"

We thank Reviewer 1 for catching the typo, and the text has been modified accordingly.
Reviewer 1: It might be more accurate to use zebrafish protein nomenclature for the transcription factor motifs in Fig1D, G and Fig2E (i.e. Gata2 not GATA2)

It is true that the currently used nomenclature is human. However, this reflects the databases used for transcription factor prediction. Currently, the best available databases for vertebrate transcription factor predictions are based on mice and humans, and the nomenclature reflects this fact.

Reviewer 2 (Review commons)
Reviewer 2: It's important where Prox1 is expressed in the lymphatic system, but if the identified enhancers also regulate Prox1 expression in other tissues like the myocardium, it would be a significant finding. Do these enhancers have a role in controlling Prox1 expression in lymphatics and non-lymphatics?

We thank Reviewer 2 for this suggestion. We have added whole-body images of GFP expression driven by *-2.1prox1a* in a 5 days post fertilization embryo (Fig. 2C). Notably, this lymphatic valve enhancer was not active in the heart or in the cardiac valves (Fig. S2E). Similarly, while the whole-body images of the remaining enhancers revealed additional domains of expression, such as the pancreas and the gallbladder (Fig. S1I, S3F-G-H), none of them showed activity in the myocardium.

Reviewer 2: Figure 4H is probably incorrect. Looking at the morphology of the valve, it's inferred that the lymph flow goes from right to left. In this case, if the valve function is abnormal, the lymph flow that entered from the right should reflux back to the left. Is the reviewer misunderstanding something here? Clarity on this point could be provided with video images, similar to echocardiograms in mice.

In zebrafish embryos the flow indeed goes from right to left direction (the left being the FCLV and right the FLV). Shin et al. (2019) has described that in the wild-type anaesthetised zebrafish embryos the valve becomes shut and not flow passes through. Therefore, none of the injected tracker should be observed past the valve position. Our experiments followed the published design and the zebrafish embryos (control and mutants) were anaesthetised with tricaine before the injections took place. We have added a schematic to Fig. 5E to better illustrate the two scenarios. We have also modified the text to explain this more clearly as we realise it could create confusion for readers: "*A correctly formed valve should be closed under tricaine anaesthesia and therefore in an anaesthetised wild-type embryo the Qtracker injected in the LFL should not leak into the FCLV. However, when the valve is not correctly formed, and cannot close properly, the Qtracker can leak into the FCLV (Fig. 5E) (Shin et al., 2019)*"

Reviewer 2: It's important where Prox1 is expressed in the lymphatic system, but if the identified enhancers also regulate Prox1 expression in other tissues like the myocardium, it would be a significant finding. Do these enhancers have a role in controlling Prox1 expression in lymphatics and non-lymphatics? (Incorporated experiment)

© 2024. Published by The Company of Biologists under the terms of the Creative Commons Attribution License (https://creativecommons.org/licenses/by/4.0/).

We have added whole-body images of GFP expression driven by the identified enhancers in 5 days post-fertilisation embryos (Fig. 2C, S1I, S3F-G-H), and we have highlighted the additional domains of expression identified.

Reviewer 2: Regarding Figure 4G, the actual image is unknown due to the red shading. Can you discuss or clarify how knocking out the Prox1 enhancer affects valve formation? If Prox1 expression is reduced, does the normal extracellular matrix change? Why do these morphological changes occur?

We thank Reviewer 2 for pointing this out. We have modified Fig. 5 by adding the unshaded version of the EM images. We have also integrated a point summarising the known regulation of the leaflet extracellular matrix by PROX1 in the discussion: "*The -2.1prox1a mutants are viable and fertile and only show developmental defects in the lymphatic valve regarding the morphology of the leaflets. As mentioned above, key factors in the regulation of the leaflet extracellular matrix such as integrin-a9 (Bazigou et al., 2009) have been suggested to be regulated under the Prox1/Foxc/Nfatc1/Gata2 network in zebrafish lymphatic valve formation (Shin et al., 2019), which could explain the observed phenotype..*"

Reviewer 2: In mice, Prox1 is expressed in the heart valve endothelium. Have they checked the enhancer activity in other valves (heart valves or venous valves, if they exist)?
Reviewer 2: -2.1prox1a is said to be important for Prox1 expression in the facial lymphatic valves. Are valves only formed in this part of the lymphatics in zebrafish at this stage? Why is it important to identify the enhancer for this particular valve? Are there no other lymphatic valves? Please explain in the text.

In the late embryonic and early larval stages of zebrafish, the facial lymphatic valve is the only lymphatic valve which has been described so far. Following Reviewer 2's suggestions, we additionally characterised the activity of the *-2.1prox1a* enhancer in the other vascular valves present in zebrafish at 5 dpf. These include two lymph- venous valves in the face as well as in the developing atrioventricular and bulboventricular cardiac valves. The results have been integrated into Fig. S2E and the text of the manuscript: "*We additionally investigated enhancer activity in other vascular valves present during embryonic development. Two lymphovenous valves (LVVs) have been described to connect the facial lymphatics to the veins (Meng et al., 2023; Shin et al., 2019). We detected weak activity of -2.1prox1a in the anterior LVV connecting the FCLV to the PHS, but none in the posterior LVV (Fig. S2D). No activity was detected in association with the developing atrio-ventricular and bulbo-ventricular cardiac valves (Fig. S2E). Whole-body imaging at 5 dpf revealed additional expression only in a population of cells in the abdomen and extremely weak expression in few muscle cells (Fig. 2C).*"

Reviewer 2: They seem to emphasize +15.2's role in facial lymphatic expression. Compared to the trunk, why is it significant that there is a unique enhancer acting in this area? Is there something functionally or anatomically unique about facial lymphatics? Please discuss why the enhancer region's conservation is high in facial lymphatics.

We thank Reviewer 2 for pointing out the missing information. We have added references in the discussion explaining the unique regulation involved in the formation of the FCLV and lymphatic valve compared with the rest of the lymphatic vasculature. "*Two of the enhancers, -2.1prox1a and +15.2prox1a, show significantly enriched expression respectively in the valve and the FCLV. These anatomically and functionally distinct subsets of the lymphatics are also regulated by different networks from the remaining lymphatics (Hußmann et al., 2023; Meng et al., 2023; Shin et al., 2019).*"

Reviewer 3 (Review commons)
Reviewer 3: Probably they should also examien a few more fish genome. I don't find this evolutioanry analysis extremely convinced and careful - which doesn't mean that the conclusions are wrong

The conservation analysis is shown in Fig. 1A (and Fig. S1B) was used to identify candidate enhancers conserved between mammals and zebrafish, and therefore included only a few Actinopterygii species. However, a second conservation analysis was performed (Fig. S4A-B) with

© 2024. Published by The Company of Biologists under the terms of the Creative Commons Attribution License (https://creativecommons.org/licenses/by/4.0/).

the objective of better understanding the conservation and/or loss of the identified *prox1a* enhancers within the actinopterygian clade. This analysis includes nine Actinopterygii species selected with a good spread within the group; however, if the reviewer would like to suggest additional fish genomes that should be included to strengthen our analysis, we would be happy to modify it.
In addition, we have added the *+15.2prox1a* enhancer to the panel, and the conservation of *-2.1prox1a* and *+15.2prox1a* within Actinopterygii has been addressed in the text: *"As expected, both -2.1prox1a and +15.2prox1a are conserved across Actinopterygii."*

Reviewer 3: They also perform genome alignment using non-duplicated outgroups (gar, xenopus) without discussing. I am a bit skeptical about the use of mVISTA on relatively short expert of sequence aroudn a gene, as it is not able to capture the global molecular evolution parameters. I think the authors should also examine some of the precomputed phastCons / phylocons data performed and available on UCSC to confirm their findings.

We thank Reviewer 3 for this comment. We have added an additional panel in Fig. S1D and Fig. S3E showing the conservation of the identified enhancers across vertebrates and actinopterygians using PhyloP and the Multiz vertebrate alignment tracks on UCSC. The text has been modified accordingly:
"The conservation of the identified elements was further confirmed using PhyloP and the Multiz alignment to vertebrate tracks in the UCSC Genome browser (Fig. S1D)" "The reduced level of conservation of -87prox1a and -14prox1a also explains why only -71prox1a is marked as conserved in the UCSC Genome Browser Multiz Alignment (Fig. S3E)"

Reviewer 3: I find the presentation, fairly obscure, the writing is quite convoluted, and the figures are very dense and not super explanatory, I would urge to improve (this is not helped by the fact that figure are their leged and presented at distinct places of this manuscript). For instance, I think having. a figure summarising signal from evolutionary conservation, scATAC and chromatin marks altogether would be quite essential.

We thank Reviewer 3 for these observations. We have modified our figures by adding titles to the different subsections, which we hope has increased the readability of the described results. We have also inserted a summarising panel showing in one place all the relevant data for the five enhancers we identified in Fig. S1C. The panel includes the snATAC signal, evolutionary conservation analysis, and available chromatin markers. However, no good lymphatic (nor endothelial) histone modification database is currently available, and therefore the represented data refer to whole-body data from earlier stages. For this reason, H3K4me1 (marking primed enhancers) was considered together with H3K27ac, which marks active enhancers.

Reviewer 3: I also find the reanalysis of the single-cell ATAC described too scarcely: which are the genes used to identify the different cell populations

We have added more details in the methods section about how the different populations have been identified in the scATAC analysis: *"Briefly, the LEC cluster was defined by combined high accessibility at the prox1a, cdh6, and lyve1b loci. The VEC clusters by combined high accessibility at the cdh5, kdrl, and stab2 or lyve1b loci, and AECs by combined high accessibility at the cdh5, kdrl, flt1, and dll4 loci but low accessibility at lybe1b. MuLECs were identified based on the high accessibility at osr2 in addition to the standard LEC loci but low accessibility at cdh6, and the endocardium by the accessibility at the cdh5 and hand2 loci."*

Reviewer 3: I also feel that the evolutionary aspect could be discussed a bit more, what are the differences between the diffeerent vertebrate lineage, etc...

We have added a passage in the text that addressed the known molecular similarities in the lymphatic development of zebrafish and mammals, such as growth factor signalling and transcription factors, as well as the obvious molecular differences, such as the presence of only one valve in zebrafish, which highlights how interesting it is that we found such strongly conserved lymphatic valve enhancers. *"Despite the obvious morphological differences, the molecular program underlying LEC development in mammals and zebrafish presents strong similarities, such as the role of growth factors signalling and TFs such as PROX1, NFATC1 or GATA2 (Kazenwadel et*

© 2024. Published by The Company of Biologists under the terms of the Creative Commons Attribution License (https://creativecommons.org/licenses/by/4.0/).

al., 2015; Koltowska et al., 2015a; Norrmén et al., 2009; Secker and Harvey, 2021; Shin et al., 2019; Wigle and Oliver, 1999). Therefore, it is worth noticing that the majority of the binding sites for these conserved lymphatic regulators are found in the sequence conserved enhancers."

Reviewer 3: (p7) active enhancer in a tissue: while ATAC gives a good indicated of accessibility it is not an indicate of activity as for instance H3K27Ac would be.

We agree with Reviewer 3 that specific histone modifications (such as H3K4me1 or H3K27ac) are more reliable indicators of enhancer identity than simple chromatin accessibility. However, no dataset mapping such histone modifications in lymphatic or endothelial cells in zebrafish exists owing to technical limitations. Therefore, in this study, we used the best available method (snATAC), which allows the identification of both enhancers and silencers. In the Results section, we clearly explain why we were limited in using histone modifications as a proxy for enhancer identity: "*Histone modifications have been linked to non-coding elements such as promoters and enhancers. Specifically, H3K4me1 marks primed and active enhancers (Heintzman et al., 2007), whereas H3K27ac marks active enhancers (Bonn et al., 2012; Creyghton et al., 2010). Currently, no LEC-specific zebrafish databases are available for H3K4me1 and H3K27ac. Whole-body databases were instead used to identified ten of the selected prox1a CNEs as primed enhancers*"

Reviewer 3: The authors did not discuss the fact that euteleosts underwent an extra whole genome duplication and that prox1a might have a paralogue.

We apologise for the lack of clarity. Fig. S1B shows the analysis of the gene that has been previously identified in the literature as the *prox1a* paralogue, called *prox3* or *prox1b*. Briefly, this figure shows the sequence conservation analysis, which did not reveal any promising candidates.

Reviewer 3: I feel the one additional experiment that the authors could have done would have been to use their construct to isolate the different cells population of interest and perform some regulatory profiling scuh as ATAC-seq or cut-and-tag on this population, to have a direct, in situ evidence of the activity of these regulatory elements.?

As discussed in the initial rebuttal, such an experiment would doubtlessly provide important insight into the specific molecular signature associated. For example, it would allow the comparison between the valve-forming cells and regular lymphatic endothelial cells, and the lines we generated in this study would be great tools to answer these questions. However, the goal of this study was to identify *prox1a* enhancers responsible for driving the expression of this key factor in the lymphatic endothelium. Nonetheless, we would be glad to provide the vectors we generated to any laboratory interested in running such an experiment, and we do not exclude that we might look into these aspects ourselves in the future.

Additional Reviewer appointed by Development (Reviewer 1)

Reviewer 1
Comments for the Author: The studies as described are relatively preliminary, in terms of utilising existing databases to identify conserved cis-acting sequences, revealing lymphatic reporter gene expression in distinct vessel beds and a potential function for a 102 bp sequence in lymphatic valve development.

The study does not really address its stated central aim, to identify the upstream regulation of prox1a in lymphatics. Each of the identified cis-regulatory elements, and most notably the -2.1prox1a element, is assigned a series of putative TF binding sites, predicted by in silico analyses, many of which have been implicated in vessel development. These TFs are not tested in the context of directly regulating prox1a expression, via a more site-specific mutagenesis and/or direct targeting of TFs using a LEC-conditional deletion approach.

We thank Reviewer 1 for their constructive feedback on our manuscript. We agree that our paper does not shed light on novel TFs regulating *prox1a* expression in the lymphatic endothelium. However, the goal of our study was to identify the previously unknown cis-regulatory elements responsible for *prox1a* regulation and characterize their activity and, in the case of *-2.1prox1a*,

© 2024. Published by The Company of Biologists under the terms of the Creative Commons Attribution License (https://creativecommons.org/licenses/by/4.0/).

function. The identification of the TFs binding to the enhancer elements, as well as the direct targeting of TFs using a LEC- conditional deletion approach, would reveal important insights into the regulation of LECs identity and lymphatics network development, but these types of experiments are technically challenging in the zebrafish model and therefore we considered them outside of the scope of our paper.

However, to clarify the aims of our study and to avoid misleading the reader we have modified the text to emphasise that we focus on identification of the enhancer sequences that can drive *prox1a* expression in the LECs. Such alterations include: "*the upstream cis-regulation of PROX1 remains largely unexplored (…) However, the cis-regulatory elements of prox1a and their evolution are still to be described. (…) In this study, we aimed to characterise the enhancers regulating the expression of prox1a (…)*"

As such the study falls short of providing insights into the molecular regulation of prox1a and identifying specific pathways that may regulate its function(s) across lymphatic vascular beds. In addition, spatial resolution is lacking with regards to whether the elements identified, and putative regulatory TFs influence LEC fate versus lymphatic vessel formation, or morphogenesis.

Our study has focused on functional studies of one of the identified conserved enhancers. Our mutant analyses have revealed that this enhancer is necessary to set up correct levels of Prox1 expression in the valve (Fig. 5A) which has been suggested in the literature (Johnson et al, 2008) to have a role in maintenance of LEC identity. In addition, we showed that this enhancer element is required for proper morphogenesis of the valve. In order to begin dissecting the transcriptional network and the relevance of the predicted TF binding sides in the identified conserve enhancers we have performed two addition experiments. First, we have assessed if the levels of *+15.2prox1a* expression were affected by the loss of Mafb transcription factors *(Fig. 1E,* please see the detailed response in the Reviewer 1 (review commons), comment 1). In addition, as suggested by the reviewer, we have mutated the putative NFATC1 binding site in the *-2.1prox1* enhancer (Fig. 2E, for detailed responseplease read the last comment in this review section).

Finally, extrapolating from ray-finned fish the relevance to mammalian Prox1 regulation is unclear; the authors describe some microsynteny, but do not test these regions in the context of mammalian (eg. mouse) conservation.

In our study, we have idented two conserved enhancers. One of them, *-2.1prox1a,* has previously been characterised in mouse (where it is called *-11Prox1*), where it is enriched in the lymphatic valves (Kazenwadel et al, 2023). Moreover, the mouse enhancer tested in zebrafish drives expression in the facial LECs, showing the functional conservation of these enhancers, and highlighting the relevance of interspecies studies. The additional enhancers (*-87prox1a*, *-71prox1a*, *-14prox1a*) were identified using chromatin accessibility on zebrafish sorted LECs and were not conserved in mammals, showing unsurprisingly that while part of the regulation of *prox1* expression is conserved between zebrafish and mammals, lineage specific divergence has also taken place.

Reviewer 1: Figures 1-4 and relevant Supp Figures: inclusion of low-resolution images would be helpful to orientate the high-resolution panels (C. E and F); in terms of anatomical localisation of the reporter expression relative to the whole embryo at the developmental stages shown.

We thank Reviewer 1 for the suggestion. We had added tile-scans of whole-body embryos at 5 dpf (Fig. 2C, Fig. S1I, S3F-G-H), which will help the reader in identifying the location of the described lymphatic vessels.

Reviewer 1: Figure 4G: the EM images show dysmorphic valves in delta-2.1prox1a mutants, but it is unclear on the extent of variation here and penetrance of phenotype(?). In addition, do the authors have a view as to whether the phenotype is underpinned by shorter cell length or is this a morphogenesis phenotype? More importantly, what do the authors think is the role of prox1a specifically in this context of valve development? Some speculation in the Discussion here would be a useful addition.

We thank Reviewer 1 for the comment. In Fig. 5C we report the percentage of observed

© 2024. Published by The Company of Biologists under the terms of the Creative Commons Attribution License (https://creativecommons.org/licenses/by/4.0/).

dismorphic valves, which provide an indirect quantification of the frequency of the phenotype. At 7 and 14 dpf, we observe respectively an increase by 25% and 35% of the amount of embryos showing severe leaflet morphology disruption. The observations in Fig. 5B and Fig. S6A-E show that the morphology of the surrounding vessels is unaffected, and the observed phenotype of reduced length of the leaflet is specific for the valve.
We have added a paragraph in the Discussion addressing in more detail what is known about the role of Prox1 in valve morphogenesis, and the relevance of the examined enhancer: "*In the case of -11Prox1/-2.1prox1a, the element drives expression in the lymphatic valve in both mouse and zebrafish. Both in mouse and zebrafish, Prox1/prox1a is a key player in lymphatic valve formation (Bazigou et al., 2009; Norrmén et al., 2009; Sabine et al., 2012; Shin et al., 2019), acting in in a regulation network with Foxc1, Nfatc1 and Gata2 to activate key downstream targets for valve development such as integrin-a9 (Bazigou et al., 2009; Shin et al., 2019) and connexin-37 (Sabine et al., 2012). The conserved cluster of binding sites for Foxc, Nfatc1 and Gata2 in the -11Prox1/-2.1prox1a enhancer strongly suggests this enhancer is part of this network. Despite this, the mice and zebrafish phenotypes for - 11Prox1/-2.1prox1a mutants are profoundly different. The -2.1prox1a mutants are viable and fertile, and only show developmental defects in the lymphatic valve regarding the morphology of the leaflets. As mentioned above, key factors in the regulation of the leaflet extracellular matrix such as integrin-a9 (Bazigou et al., 2009) have been suggested to be regulated under the Prox1/Foxc/Nfatc1/Gata2 network in zebrafish lymphatic valve formation (Shin et al., 2019), which could explain the observed phenotype. Conversely, mouse -11Prox1 mutants have more severe lymphatic defects and die perinatally (Kazenwadel et al., 2023). The deletion of - 11Prox1 is fully phenocopied by the deletion of the GATA2 binding site, which is also responsible for the transition of LECs to HECs in -11Prox1 mutant mice (Kazenwadel et al., 2023). However, the putative GATA2 binding site is not conserved in zebrafish, which could again explain the different severity of the phenotype.*"

Reviewer 1: Figure 4H. The qTracker images are difficult to reconcile as having observable differences, and the explanation of these panels (with respect to valve function in sibling control v mutant fish) is lacking in the text (page 11).

We thank Reviewer 1 for pointing out the unclarity in the presentation of our experimental design and results. To make the panel easier to read, we have added a schematic representation of the injections to Fig. 5E, which will hopefully make the interpretation of the result clearer. We have moreover better addressed our experimental design and the expected results in the text: "*A correctly formed valve should be closed under tricaine anaesthesia and therefore in an anaesthetised wild- type embryo the Qtracker injected in the LFL should not leak into the FCLV. However, when the valve is not correctly formed, and cannot close properly, the Qtracker can leak into the FCLV (Fig. 5E) (Shin et al., 2019).*"

Reviewer 1: What is the functional significance of conserved regions of microsynteny of the zebrafish prox1a enhancers (most notably +15.2 prox1a and -2.1prox1a) with the regulation of mammalian/murine Prox1?

We have added a paragraph to explain the relevance of microsynteny in the *prox1a* region in vertebrates for the presence of conserved enhancers: "*Local microsynteny, which is the conservation of the identity and position of the features surrounding the gene of interest, was verified by comparing the identities of neighbouring loci (Fig. S1A). The presence of microsynteny indicate that no major genomic rearrangement, such as insertions, deletion or inversion of whole regions, have occurred around the considered locus, and therefore supports the presence of evolutionary conserved enhancers.*" In the Discussion, we have also addressed the potential interest of conserved enhancers in the identification of conserved factors of lymphatic development in mouse /humans: "*Despite the obvious morphological differences, the molecular program underlying LEC development in mammals and zebrafish presents strong similarities, such as the role of growth factors signalling and TFs such as PROX1, NFATC1 or GATA2 (Kazenwadel et al., 2015; Koltowska et al., 2015a; Norrmén et al., 2009; Secker and Harvey, 2021; Shin et al., 2019; Wigle and Oliver, 1999). Therefore, it is worth noticing that the majority of the binding sites for these conserved lymphatic regulators are found in the sequence conserved enhancers.*"

Reviewer 1: In the targeted 102 bp spanning the putative NFATC1 binding site (in the -2.1prox1a

© 2024. Published by The Company of Biologists under the terms of the Creative Commons Attribution License (https://creativecommons.org/licenses/by/4.0/).

enhancer) what is the predicted additional TF binding? How does targeting the NFATC1 site alone, versus neighbouring sequences, affect prox1a expression in the valve versus valve morphogenesis? This gets at the more general comment above and the need to define upstream TF regulation of prox1a expression/function.

We thank Reviewer 1 for suggesting this experiment. In order to address the functional importance of NFATC1 for the activity of the *-2.1prox1a* enhancer, we have injected embryos with a mutated version of the *-2.1prox1a* reporter vector in which the binding site for NFATC1 has been removed. Comparing the obtained GFP expression level in the valve with that of positive control (non-mutated enhancer) and negative control (scrambled enhancer), we could observe a reduction of 40% in enhancer activity in the GFP+ valve cells. Due to revision time constrained we could not generate stable lines.
The results have been addressed in the main text: "*In order to test the functional relevance of the predicted TFs, we focused on the conserved NFATC1 binding site. We injected embryos with the e1b TATA minimal promoter vector (Villefranc et al., 2007) containing either the WT -2.1prox1a sequence or one lacking the NFATC1 site. We compared the level of activity in the valve at 5 dpf, andobserved a 40% reduction of intensity in the mutated enhancer compared to the WT (Fig. 2E). No activity was observed in the scrambled-sequence controls (n=36). This suggests that NFATC1 is indeed needed for the full activity of -2.1prox1a, but that even in its absence other factors can drive partial expression in the valve.*"

Second decision letter

MS ID#: DEVELOP/2023/202525

MS TITLE: Multiple cis-regulatory elements control prox1a expression in distinct lymphatic vascular beds

AUTHORS: Virginia Panara, Hujun Yu, Di Peng, Karin Staxang, Monika Hodik, Beata Filipek-Gorniok, Jan Kazenwadel, Renae Skoczylas, Elizabeth A Mason, Amin Allalou, Natasha Harvey, Tatjana Haitina, Ben L Hogan, and Katarzyna Koltowska

I am happy to tell you that your manuscript has been accepted for publication in Development, pending our standard ethics checks.

Reviewer 1

Advance summary and potential significance to field

As previous review;

This study sheds light on both conserved and species-specific (zebrafish) regulation of Prox1/PROX1 expression and function in various lymphatic beds during development. This is significant in light of the role of prox1/PROX1 as the master regulator of lymphatic endothelial cell specification/identity.

Comments for the author

The authors have improved the study with text clarifications, improved figures and new data. In particular the new functional experiments: i)importance of mafba/mafbb binding, as a known lymphatic regulator, to the +15.2prox1a enhancer for regulating prox1a activity and ii) testing NKATC1 binding as required for-2.1prox1aenhancer activity in the lymphatic valve, are welcome.
No further comments.

© 2024. Published by The Company of Biologists under the terms of the Creative Commons Attribution License (https://creativecommons.org/licenses/by/4.0/).
